# Light organ photosensitivity in deep-sea shrimp may suggest a novel role in counterillumination

**DOI:** 10.1038/s41598-020-61284-9

**Published:** 2020-03-11

**Authors:** Heather D. Bracken-Grissom, Danielle M. DeLeo, Megan L. Porter, Tom Iwanicki, Jamie Sickles, Tamara M. Frank

**Affiliations:** 1 Department of Biology, Florida International University, North Miami, FL 33181 USA; 2 Department of Biology, University of Hawai’i at Mānoa, Honolulu, HI 96822 USA; 3 Department of Biology, Nova Southeastern University, Fort Lauderdale, FL 33314 USA

**Keywords:** Evolution, Zoology

## Abstract

Extraocular photoreception, the ability to detect and respond to light outside of the eye, has not been previously described in deep-sea invertebrates. Here, we investigate photosensitivity in the bioluminescent light organs (photophores) of deep-sea shrimp, an autogenic system in which the organism possesses the substrates and enzymes to produce light. Through the integration of transcriptomics, *in situ* hybridization and immunohistochemistry we find evidence for the expression of opsins and phototransduction genes known to play a role in light detection in most animals. Subsequent shipboard light exposure experiments showed ultrastructural changes in the photophore similar to those seen in crustacean eyes, providing further evidence that photophores are light sensitive. In many deep-sea species, it has long been documented that photophores emit light to aid in counterillumination – a dynamic form of camouflage that requires adjusting the organ’s light intensity to “hide” their silhouettes from predators below. However, it remains a mystery how animals fine-tune their photophore luminescence to match the intensity of downwelling light. Photophore photosensitivity allows us to reconsider the organ’s role in counterillumination - not only in light emission but also light detection and regulation.

## Introduction

Photoreceptor cells inside the complex eyes of animals are responsible for light detection and subsequent signaling cascades linked to vision. Though light detection in animals is typically associated with ocular photoreceptors, the ability to detect and respond to light can also occur in extraocular tissues and structures^1^. Extraocular photoreception has been documented across a range of structures and taxa, including the dermal chromatophores of cephalopods and fish, tube feet of echinoderms, pineal organs in fish and the central nervous systems of arthropods^2–6^. Despite the occurrence across diverse metazoans, knowledge regarding the functionality of extraocular photoreceptors remains limited.

Bioluminescent light organs, called photophores, provide a unique opportunity to study extraocular photosensitivity, as evidence suggests these structures not only emit light but can also detect it^7^. Photophores are complex organs composed of bioluminescent cells (photocytes), and sometimes pigments, reflectors, and filtering structures^8^. They can be divided into two types: bacterial or autogenic, where the light is produced by either symbiotic bacteria living within the structure or by the animal itself. In some species, photophores assist in a form of camouflage known as counterillumination. During this process, photophore emissions mimic the downwelling light blocked by the animal’s body, thereby camouflaging the animal’s profile that would otherwise be detectable to predators below^9,10^.

**Figure 1:**
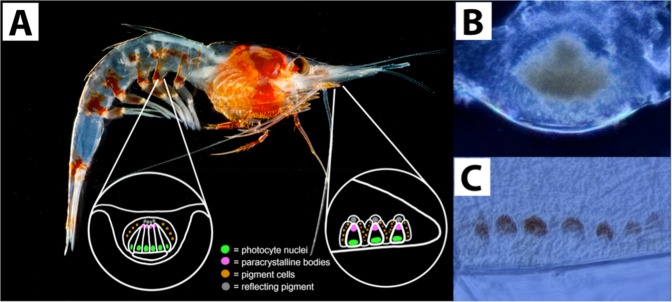
Lateral view of *Janicella spinicauda* (A. Milne-Edwards, 1883). (**A**) Dermal photophores (red dots) along the pleopod (inset, left) and maxilliped (inset, right) are expanded to illustrate the internal structures. A detailed description on the ultrastructural differences between maxilliped and pleopod photophores can be found in^8^. Light micrographs of pleopod photophores (**B**) and maxilliped photophores (**C**) are provided for context. Photo credit: Danté Fenolio.

Deep-sea shrimp of the family Oplophoridae possess autogenic photophores in three (*Systellaspis, Oplophorus* and *Janicella*) of the ten genera^11^. Within these three genera, species vertically migrate into shallower waters where counterillumination would be a useful mechanism for survival. The light organs in these species are located across the entire length of the shrimp’s body and appendages^8^. Depending on the location of the photophores, they vary slightly in structure, ranging from an individual unit consisting of a single photocyte (ex. 3^rd^ maxilliped photophores) to dense clusters of photocytes (ex. pleopod photophores)^8^ (Fig. 1). Both types contain similar underlying components including light-emitting cells (photocytes), paracrystalline bodies, reflecting pigment cells, and carotenoid pigment cells (Fig. 1). In some species, the highest densities of photophores are located ventrally along the cephalothorax and abdomen, presumably to aid in counterillumination. However, photophores are not restricted to the undersides of the shrimp and can commonly be found both dorsally and laterally along the entire length of the carapace and tail fan. More intriguingly, they can present themselves as conspicuous streaks or high-density clusters along the dorsal and lateral regions of the eyes and/or the eyestalks (per observation).

Although counterillumination has been experimentally demonstrated in vertically-migrating shrimp^12^ and other marine species^13,14^, it remains a mystery as to how animals fine-tune their photophore luminescence to match the intensity of downwelling light. As animals vertically migrate through the water column or as light intensity changes with photic period, the control of photophore emissions for effective camouflage is crucial. This fine-tuning of counterillumination has been experimentally shown in deep-sea shrimp, where the shrimp can alter the intensity, angle and spectral distribution of the light emitted by the photophore^15^. Increased visual sensitivity of photophore-bearing shrimp has been suggested as a possible mechanism for emission matching^16^, however anatomical investigations of the eyes and light absorption models refute this hypothesis^17^. Here, we test for extraocular photosensitivity in the bioluminescent light organs of deep-sea shrimp and find compelling evidence for light sensitivity through three independent lines of research. Photophore photosensitivity allows us to reconsider the organ’s role in counterillumination - not only in light emission but also light detection and regulation.

## Results

In this study, we use transcriptomics, *in situ* hybridization, immunohistochemistry and shipboard light-exposure experiments to test photosensitivity in the autogenic light organs of *Janicella spinicauda*. Live specimens were collected without exposure to light aboard the *RV Walton Smith* from the Florida Straits. Our first approach used RNA sequencing (RNAseq) and phylogenetic methods to search for opsins and phototransduction genes in the photophores, both of which are known to play a role in light detection in most animals^18,19^. Our second approach incorporated *in situ* hybridization and immunohistochemistry methods to investigate tissue-specific expression of the opsin and phototransduction genes identified in the RNAseq studies. Lastly, we used transmission electron microscopy to determine if changes occurred in specific organelles, present in photophores and known to be light sensitive in photoreceptors, in response to dim and damaging levels of light exposure.

### RNAseq

An average of 29.3 M reads were generated per sample. These data are available on the NCBI’s Sequence Read Archive database under Bioproject ID: PRJNA521050. The quality assessment of the tissue-specific *de novo* transcriptomes revealed 140,357 and 201,159 contigs with a mean length of approximately 617 and 782 base pairs (bp) for the eye and photophore assemblies, respectively (Supplementary Table 1). N50 statistics to evaluate assembly contiguity were based on the longest isoform per putative gene and were 908 bp (eye) and 1356 bp (photophore). For the eye reference assembly 69.5% of universal single-copy arthropod orthologs were identified (Complete [C]:61%, Single [S]:51.1%, Duplicated [D]:9.9%, Fragmented [F]:8.5%, Missing [M]:30.5%, n:1066) compared to 84.7% identified in the photophore assembly (C:78.8%, S:61.6%, D:17.2%, F:5.9%, M:15.3%, n:1066). The BUSCO scores reflect the specificity of the targeted tissue-specific transcriptomes, though they indicate relatively high-quality assemblies. It is possible a portion of missing BUSCOs is a result of divergent or complex gene structures^20^ and/ or technical limitations (i.e., gene prediction), which were shown to inflate proportions of fragmented or missing BUSCOS for large genomes. Given the large estimated genome size for crustaceans this may also be a contributing factor.

**Figure 2:**
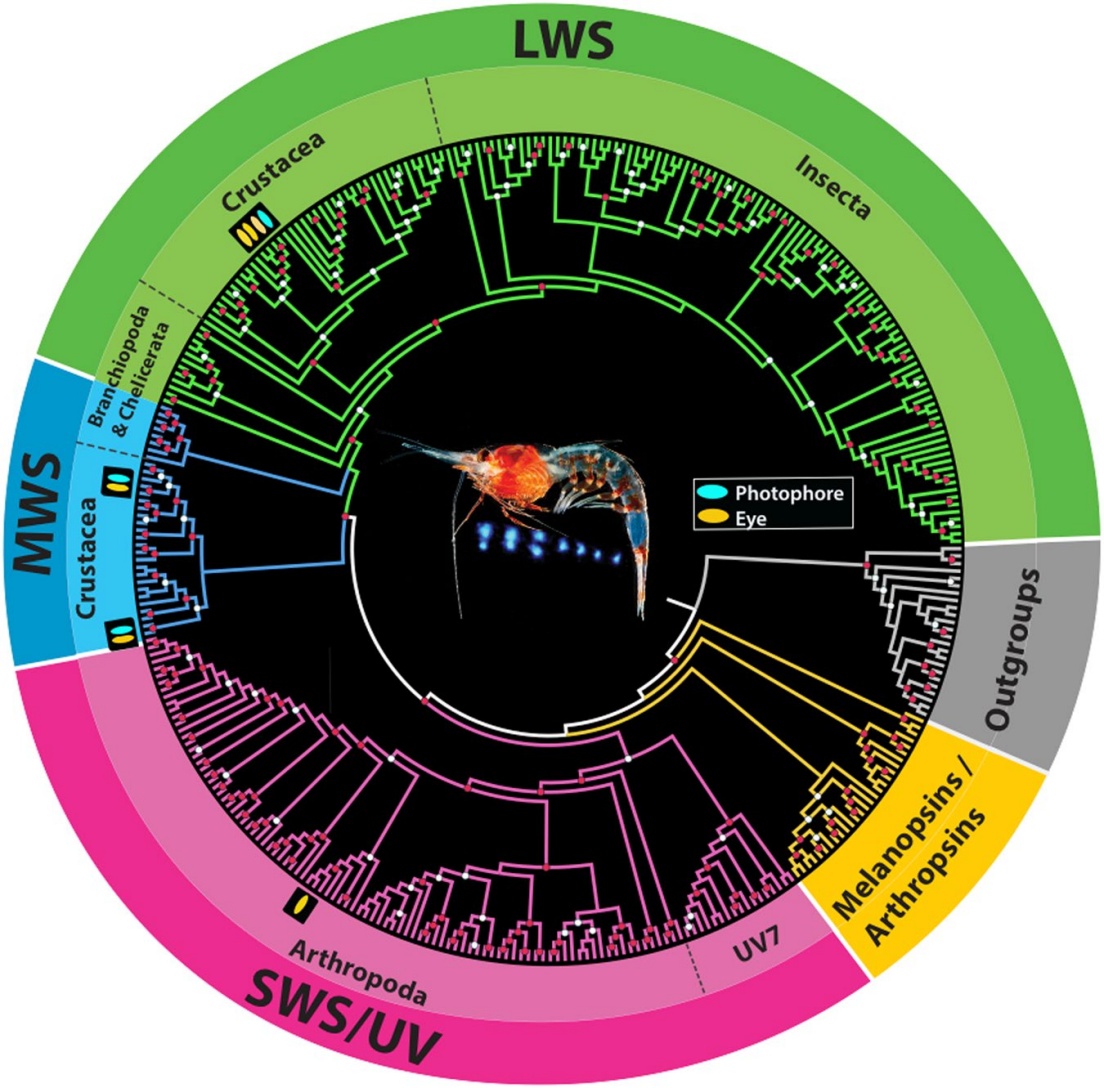
Phylogenetic opsin tree reconstruction comprising 325 rhabdomeric opsins (r-opsins) and closely related melanopsins/arthropsins. Newly curated r-opsins from the *J. spinicauda* photophore (blue oval) and eye (orange oval) transcriptomes were aligned with reference opsin datasets^18,50^ comprising visual opsins across a range of measured spectral sensitivities as well as non-visual opsins and related G-protein coupled receptors (GPCR). The putative spectral sensitivities of the r-opsin clades (SWS/UV = short wavelength; MWS = mid-wavelength; LWS = long wavelength) were inferred from these datasets. Significant triplicate bootstrap support is indicated by red circles (SH-aLRT >80, aBayes >0.95 and UFBoot >95) and significant duplicate bootstrap support is indicated by white circles (SH-aLRT >80 or UFBoot >95, and aBayes >0.95). Refer to Supplementary Fig. 1 phylogeny for more information. Photo credit: ©DantéFenolio. A recreation of *J. spinicauda* emitting light during counterillumination.

Phylogenetic analyses of the *J. spinicauda* photophore transcriptome revealed visual opsins belonging to two medium-wavelength sensitive clades (MWS1, MWS2) and one long-wave sensitive clade (LWS2) (Fig. 2, Supplementary Fig. 1). Nearly identical opsins (=97–100% protein similarity) were recovered from the eye transcriptome belonging to the MWS1, MWS2, and LWS2 clades, as well as a short-wavelength sensitive clade (SWS2) comprising putative ultraviolet (UV) shifted opsins. LWS2 expression was highest across both tissue types. In the photophores, LWS2 expression was 6.2X greater than MWS2 expression and 11.3X greater than MWS1 expression. These expression differences were even more prominent in the eyes (see Supplementary Table 2). Structural amino acid alignments of photophore and eye opsins revealed conserved elements characteristic of rhabdomeric opsins (r-opsins, Supplementary Fig. 2). These included the 7 transmembrane domains, two conserved Cys residues, a conserved Lys residue critical for Schiff base formation with the chromophore, an amino acid triplet similar to the one known to couple to G-alpha_q_ (HP(R/K)) and the “R(E/D)QAKKMN” sequence conserved among arthropod opsins (Supplementary Fig. 2). Phototransduction pathway analyses of the photophore assembly also identified major pathway components including the r-opsins, which initiate the signaling cascade, pathway regulators such as Gq-proteins, and the cascade terminators- retinal degeneration (rdg) and arrestin (Arr) (Table 1). These same genes were recovered from the eye assembly along with the calcium ion (Ca^2+^) channel- transient receptor potential (trp). With the exception of Phospholipase C (PLC), PIA phototransduction pathway annotations for the photophore and eye transcriptomes correspond to the same genes.

**Table 1:** Phototransduction pathway genes, annotated with Phylogenetically Informed Annotation (diamond), from *J. spinicauda* eye and photophore transcriptomes.

Gene	Eyes	Photophores
Arrestin (Arr)	♦	♦
Diacylglycerol kinase (DAGK)	♦	♦
G protein-coupled receptor kinase 1 (GPRK1)	♦	♦
G protein-coupled receptor kinase 2 (GPRK2)	♦	♦
G-alpha_q_ (Gqα)	♦	♦
G-beta_q_ (Gqβ)	♦	♦
G-gamma_q_ (Gqγ)		
Rhabdomeric opsin (r-opsin)	♦	♦
Protein kinase C (PKC)	♦	♦
Phospholipase C (PLC)	♦	♦
Retinal degeneration B (rdgB)	♦	
Retinal degeneration C (rdgC)	♦	♦
Transient receptor potential (trpl)	♦	

### Fluorescent *in situ* hybridization and immunohistochemistry

**Figure 3:**
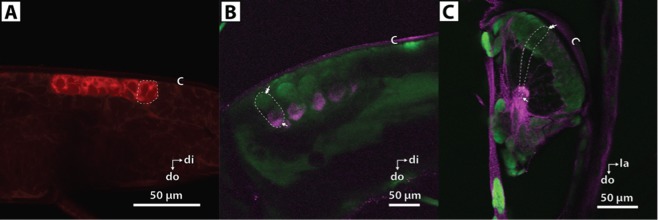
Expression of LWS2 opsin and Gq proteins in maxilliped (whole mount) and pleopod (100 μm thick sections) photophores of *J. spinicauda*. (**A**) LWS mRNA labeling is observed throughout the maxilliped photophore cell bodies. (**B**,**C**) Gq protein is localized in the apex of photophore cells in the proximity of the luminescent paracrystalline bodies. Gq protein (magenta) and LWS mRNA (red) are labeled with Cy5; cell nuclei (green) are labeled with diamidino-2-phenylidole (DAPI); tissue autofluorescence is also present (diffuse red and green). Single arrowheads point to apex of individual photocytes containing paracrystalline bodies and double arrowheads point to photocyte nuclei in the distal region of photophores. Individual photocytes are outlined with dashed white line (**A**–**C**). (*di* distal, *do* dorsal, *la* lateral, *c* cuticle). Note: Dorsal oriented downward. Refer to Fig. 1. for photophore structure and light microscopy images.

*In situ* hybridization and immunohistochemistry methods were used in the photophores to investigate tissue-specific expression of the photophore LWS2 opsin and Gq-alpha proteins known from the phototransduction cascade. LWS2 opsin expression and G-protein localization corroborated findings from the RNAseq analyses. LWS2 opsin labelling was detected in the maxilliped photophore cell bodies (Fig. 3A) indicating expression localized specifically to the photophores and not the surrounding tissue. The possibility of endogenous autofluorescence was ruled out by imaging unlabeled photophores. Labeling by anti-Gq was present in both the maxilliped photophores (Fig. 3B) and pleopod photophores (Fig. 3C). The anti-Gq signal in pleopods and maxilliped photophores was most intense near the apex of each structure, the region where luminescent granules of the paracrystalline bodies are located. The DAPI stain labeled the photocyte nuclei, which are located near the cuticle. Controls for labelling experiments showed low background (ISH) or no non-specific binding of the secondary antibody (IHC) (Supplementary Fig. S3).

### Shipboard light exposure experiments

**Figure 4:**
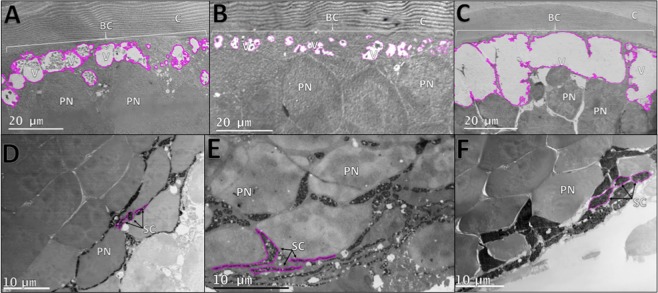
Shipboard light-exposure experiments on *J. spinicauda* photophores. Dark control (**A**), dim light exposed (**B**), and bright light exposed (**C**) photophore basal cytoplasm and vacuoles (outlined in pink). Tissue exposed to bright light exhibited a significant increase in basal cytoplasm and vacuole areas. However, dim light exposed tissue did not have a significant increase in basal cytoplasm area and had significantly less vacuolated area compared to the controls. Dorsal sheath cells (outlined in pink) in control (**D**), dim light exposed (**E**), and bright light exposed (**F**) photophores. Sheath cells from bright and dim light exposed tissues were significantly wider than those from control tissues. (*C* cuticle, *V* vacuole, *BC* basal cytoplasm, *PN* photocyte nuclei, *SC* pigment sheath cell).

**Figure 5:**
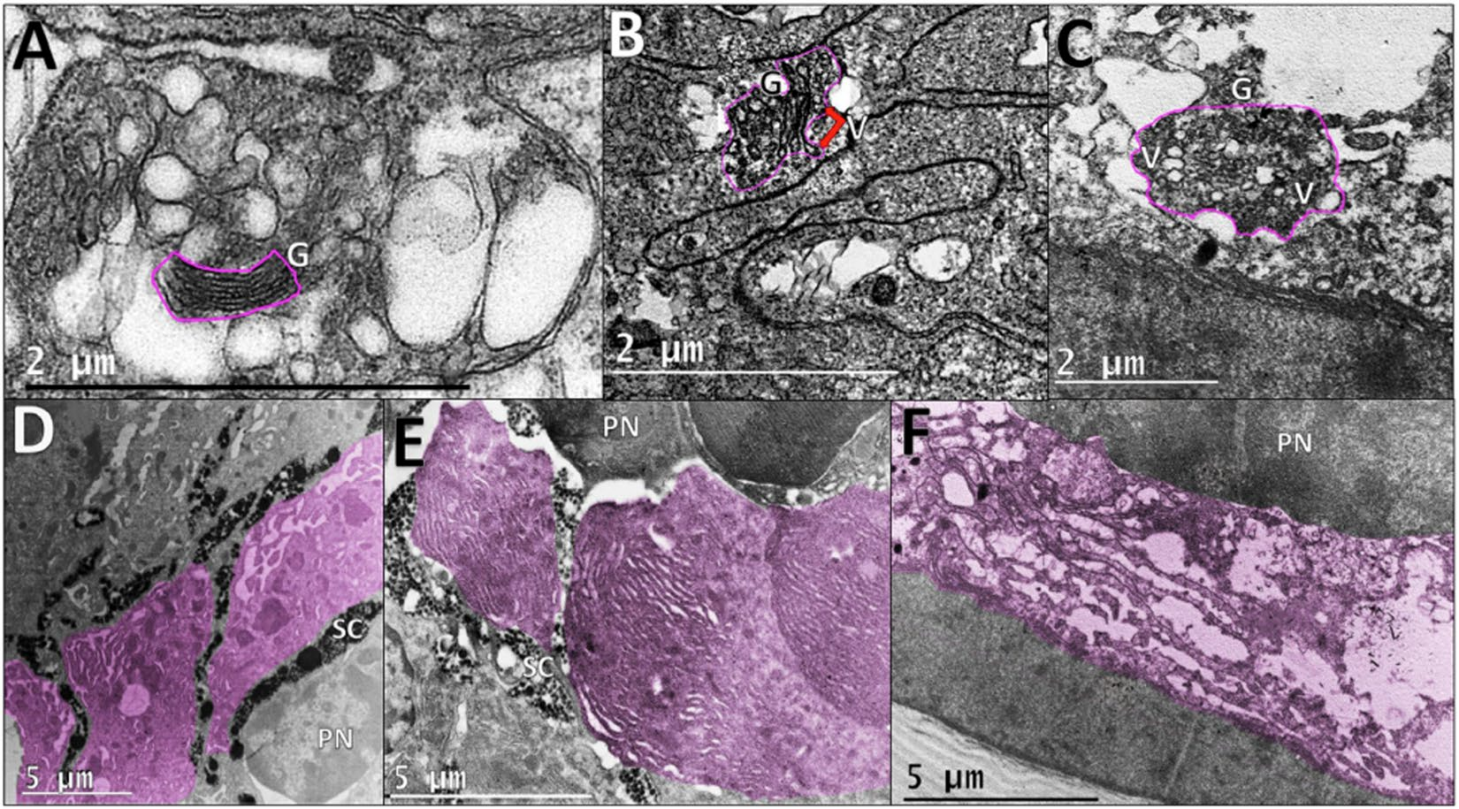
Shipboard light-exposure experiments on *J. spinicauda* photophores. Golgi bodies (outlined in pink) in control (**A**), dim light exposed (**B**), and bright light exposed (**C**) photophores. Golgi bodies had no vesiculation in the controls, were moderately vesiculated at Golgi ends (red arrow) in dim light exposed tissue* and were highly vesiculated in bright light exposed tissue. Endoplasmic reticulum (ER) (pink shading) in control (**D**), dim light exposed (**E**), and bright light exposed (**F**) photophores. ER had a dense arrangement and was less defined in the controls, was compact but well-defined in dim light exposed tissue, and was fragmented and loosely arranged in bright light exposed tissue. (*PN* Photocyte nuclei, *G* Golgi, *V* vesicle, *SC* sheath cell) *Images of Golgi bodies from tissue exposed to dim light for 60 minutes were not suitable for publication, and therefore an image from tissue exposed to dim light for 30 minutes is used to represent Golgi, as the size and appearance is consistent between timed trials.

RNAseq and localization studies indicate the presence and expression of light sensitive proteins in photophores. Therefore, shipboard light “dim” and “bright” exposure experiments were conducted on live *J. spinicauda* to further investigate photophore photosensitivity. Ultrastructure changes were observed in the basal cytoplasm, pigment sheath cells, endoplasmic reticulum (ER), and Golgi apparatus organelles of the photophore, with greater changes resulting from brighter light exposures, similar to what has been observed in the eyes of crustaceans in response to varying levels of light^21,22^. Exposure to the bright light induced significant expansions of both the basal cytoplasm and vacuole size compared to dark controls (Fig. 4A–C, Supplementary Table 3). Under exposure to dim light, there was a small decrease in the area of basal cytoplasm compared to dark controls, as well as a statistically significant decrease in the size of the vacuoles (Fig. 4B, Supplementary Table 3). Both bright light and dim light exposure elicited a conformational change in the photophore sheath cells to a more rounded, compact arrangement, relative to the linear morphology of unexposed control cells (Fig. 4D–F). The increase in sheath cell width under bright light was almost twice as much as under dim light, but both were significantly wider than in the dark controls (Supplementary Table 3). Lastly, Golgi bodies and ER were present as discrete structures without vacuolization in control photophore tissues; upon exposure to dim light, small vacuoles are present in the Golgi, and upon exposure to bright light, both organelles increased in size and abundance, and the Golgi bodies were filled with vesicles (Fig. 5A–F).

## Discussion

Light organs, such as photophores, can be divided into two types: bacterial and autogenic^15,23^. Unlike the autogenic system examined here, bacterial light organs rely on symbionts to produce luminescence. Light detection has been demonstrated in the complex bacterial light organs of the squid, *Euprymna scolopes*, suggesting this may aid the squid in managing symbiont load and/or in comparing ambient light and symbiont light emission during counterillumination^7^. Here, we used a multi-disciplinary approach to provide evidence that autogenic light organs of deep-shrimp also have light-detecting abilities. Our study is similar to that of Tong *et al*.^7^ in that we are finding a long-wavelength sensitive opsin (LWS2) in the photophore that is nearly identical to those found in the eye. However, two additional opsins with putative sensitivity to mid-wavelength light (MWS1, MWS2) were also detected in shrimp photophores, which could have implications in sensing their own emissions during counterillumination (see more discussion below). Similar to the LWS opsin, these MWS opsins are identical to those in the eyes. This high diversity of visual opsins in the photophore was unexpected, though their classification is supported by multiple lines of evidence including putative functional identifications using Phylogenetically Informed Annotation (see Methods, Fig. 2, Supplementary Fig. 1) and the presence of conserved amino acid motifs characteristic of visual r-opsins (Supplementary Fig. 2). Further, all major genes involved in phototransduction, the visual pathway in which light is converted to electrical signals in photosensitive tissues, were recovered (Table 1), with the exception of rdgB and trp. The absence of rdgB and trp from the photophore transcriptome may be due to the lack of expression at the time of sampling or fundamental differences in phototransduction signaling pathways, as they are not currently known for deep-sea shrimp. Though the presence of these genes does not confirm functionality- gene localization, expression and shipboard experimentation lend further support. Localization studies confirmed visual opsins (LWS) and phototransduction proteins (Gq) are expressed within the photophores and not in surrounding tissues (Fig. 3, Supplementary Fig. 3), while shipboard experimental exposures revealed light-induced changes to photophore ultrastructure (Figs. 4, 5) similar to what has been observed in photoreceptor tissue. The increased vacuolization evident in the basal cytoplasm of photophores exposed to bright light, together with smaller effects in dim light exposed tissue, was similar to what was seen in previous studies of the eyes of the rock lobster *Jasus edwardsii*^24^, the spider crab *Libinia emarginata*^25^, and the isopod *Cirolana borealis*^21^ (Fig. 4A–C). Moreover, these photophores contain carotenoid and ommochrome pigments in sheath cells^8^, the same pigments that are present in crustacean eyes. In crustacean eyes, these pigments migrate in response to light as a mechanism to protect retinular cells, the site of phototransduction^26^. In the current study, photophore pigment cell migration is indicated by light-induced changes in sheath cell morphology. Light exposure elicited a conformational change in the photophore sheath cells to a more rounded, compact arrangement, with the effects of bright light substantially greater than those of dim light. This suggests the onset of light triggers the photophore dorsal sheath cells to cluster in proximal regions to shield photosensitive structures, with greater effects due to bright light, as one would expect in photosensitive tissue. Lastly, both the number and size of the Golgi bodies increased in response to bright light, and these structures were severely vesiculated (Fig. 5A–C). This is similar to what has been described in photoreceptors from dim light environments. Golgi bodies are present in the light sensitive structures of crustacean eyes^26–28^ and while increased numbers of Golgi bodies are associated with normal membrane turnover processes^21,29–31^, the increase in abundance, size, and vesiculation in response to light is associated with degeneration produced by exposure to damaging light intensities^21^. Findings from shipboard experiments, combined with transcriptomic, gene expression and localization results provide compelling evidence that autogenic light organs of invertebrates play a role in light detection. Our study raises the exciting possibility that light organ photosensitivity is ubiquitous across diverse taxa and should be investigated in other bioluminescent taxa. Because light organs can be found within several lineages across the animal Tree of Life^7,23^, results from this study have the potential to reach far beyond our model system.

It has long been known that, in some species, photophores play a critical role in counterillumination as the structures responsible for the emission of light^10,12^. However, very little is known about the mechanism by which counterilluminating animals can fine-tune their emissions to match the intensity of downwelling light. Photophore-bearing shrimp, such as *J. spinicauda*, are known to have increased visual sensitivities (i.e., blue-green and near-UV visual pigments) when compared to non-photophore bearing oplophorids^16,32^. It has been suggested that this increase in visual sensitivity would allow for contrast enhancement and may aid in fine-tuning photophore luminescence^16^. Gaten, *et al*.^17^ argued, for this to be the case, rhabdoms in the dorsal part of the eye would need to be structurally modified to be efficient at detecting the contrast between downwelling light and photophore emissions^17^. Their model, which measured light absorption across the dorsal and ventral regions of oplophorid eyes, showed that the dorsal eye retained the structure seen in shallow-water shrimp (high resolution and sensitivity to downwelling light) and only the ventral eye was modified for enhanced contrast^17,33^. They concluded oplophorid visual systems are likely not responsible for photophore emission matching^17,33^ and alternative mechanisms should be explored.

With evidence for light organ photosensitivity and their positioning across both the eyes/eyestalks and length of the body, it is possible photophores are playing a larger role in counterillumination than previously thought. Light sensitivity may allow adjacent photophores to detect their *own* emissions, and the diversity of putative opsins recovered in the photophore (LWS2, MWS1, MWS2) suggests a possible mechanism for enhanced contrast detection to discriminate between photophore emissions and background light^17,34^. It is less likely, but still plausible, that the more dorsally located eye, eyestalk, and body photophores are working in conjunction with the eyes to detect downwelling light during vertical migrations. Past investigations in lantern fish have discovered eye photophores are oriented and emit light toward the eye to potentially aid in a feedback mechanism to regulate light output^35^. Whether a similar system exists in deep-sea shrimp remains unknown. Here, we suggest the most probable scenario for emission-matching involves the visual system (eyes) working in conjunction with the light-sensitive photophores to detect and mimic downwelling light, although the exact mechanisms for this feedback loop are still to be discovered. If photophores are able to detect their own emissions, coupled with eyes that are dorsally adapted to detect downwelling light^17,33^, this provides a compelling framework to further explore how animals fine-tune counterillumination. Future studies that target the neural connections of the photophore and eyes alongside behavioral and physiological studies could “illuminate” the larger role photophores play in the deep sea.

## Materials and Methods

The study of photophore photosensitivity in deep-sea species requires that the animals be protected from damaging surface light^32,36^ and warm water temperatures^37^. In this study, live specimens of *Janicella spinicauda*, a mesopelagic (200–1000 m) crustacean with cuticular photophores, were collected aboard the *R/V Walton Smith* using a light-tight, thermally insulated cod-end that could be closed at depth^32^, sorted using dim red light, and maintained at temperatures between 9–10 °C inside Koolatrons. *Janicella spinicauda* has a vertical distribution that range from ~100–1500 m in the Gulf of Mexico and Florida Straits^38^.

### RNA-sequencing

Live specimens (n = 5) were preserved in RNAlater and stored at −80 °C. Eye and photophore tissue replicates were carefully dissected and homogenized in TRIzol reagent (ThermoFisher Scientific). Total RNA was discretely extracted from tissues and rDNase (Macherey-Nagel) treated following recommendations in^39^. Libraries were prepared from isolated mRNA using the NEBNext Ultra II Directional RNA Library Prep Kit for Illumina. Barcoded libraries were size selected (Pippin Prep, Sage Science) and sequenced across an Illumina HiSeq 4000 lane. Raw sequencing data was quality assessed using FastQC^40^ to inform quality and adaptor trimming with Trimmomatic v0.36^41^. Trimmed reads were processed with Rcorrector^42^ and BBnorm to error-correct reads and normalize read coverage. Trinity v2.6.5^43^ was used to assemble tissue-specific (eye and photophore) reference assemblies *de novo* (minimum contig length of 200 bp, k-mer size of 23). Contamination was removed from each assembly using Kraken v1.0^44^ with default parameters and NCBI’s (Refseq) bacteria, archaea and viral databases. Contaminate free assemblies were then passed through BBduk and dedupe (BBTools suite, available at: http://sourceforge.net/projects/bbmap) to remove duplicate transcripts and rRNA. Transcriptome quality and completeness for each tissue-specific reference assembly was assessed using Transrate v1.0.3 and BUSCO v3.0.2 (Benchmarking Universal Single-Copy Orthologs;^45,46^. BUSCO evaluations were done in an evolutionary context using a reference dataset of orthologous groups (n = 1066) found across Arthropoda^20^. Voucher specimens (HBG 8616–8620) were ultimately curated in the Florida International Crustacean Collection (FICC).

Each tissue-specific transcriptome was analyzed using the Phylogenetically-Informed Annotation (PIA) tool^47^, modified for command-line use^48^, which characterizes putative visual opsins and phototransduction pathway genes in a phylogenetic context. Assemblies were run through PIA using a pipeline previously described in Pérez-Moreno *et al*. 2018. In brief, these tools extract all open-reading frames (ORFs), identify visual genes via BLAST-searches against a database of known visual genes, aligns and subsequently places significant hits into precomputed gene phylogenies to differentiate between false positives and genes of interest. Opsin diversity was further characterized for each tissue-specific assembly. Putative opsin sequences were aligned with PROMALS3D^49^ using a reference opsin dataset (n = 996^18,50^,) that comprises visual opsins across a range of spectral sensitivities as well as non-visual opsins and related G-protein coupled receptors (GPCR) (see Supplementary Table 4 for a list of all taxa included). Following model testing, opsin phylogenetic tree reconstruction was done with IQ-TREE^51^ using an LG general amino acid replacement matrix, under a FreeRate model with 9 rate categories, and empirical base frequencies (LG + R9 + F). Support was assessed in triplicate by (1) a Shimodaira–Hasegawa–like approximate likelihood ratio test (SH-aLRT; 10,000 replicates), (2) an approximate Bayes test and (3) an Ultra-fast bootstrap approximation (UFBoot; 10,000 replicates)^52–54^. False positives aligning with non-visual opsins or outgroups were removed. Visual opsin identity was further confirmed via structural alignments (PROMALS3D) to Bovine rhodopsin (2.8 Å) template (IF88.pdb)^55^ and the subsequent identification of conserved domains, motifs and residues characteristic of invertebrate r-opsins (as described in^56^). Structural alignments were visualized with ESPript^57^ using default parameters and a sequence similarity score based on the percentage of equivalent residues at each position in the alignment (considering the physico-chemical properties of the residues). As invertebrate and vertebrate opsins formed distinct clades, for simplicity the final phylogenetic reconstruction contains only invertebrate visual rhabdomeric opsins or r-opsins and closely related melanopsins (see Supplementary Table 4 for additional information).

Opsin expression patterns were further evaluated using the transcript quantification tool Salmon^58^ and Trinity’s standardized protocol for alignment-free abundance estimation methods^59^. Briefly, transcript per million (TPM) abundance estimates were generated for all genes using Salmon^58^. Absolute abundance measures were then generated from the TPM values using Trinity and the Trimmed Mean of *M-values* (TMM) normalization method^60^. This widely used normalization method uses a weighted trimmed mean of the log expression ratios to estimate scaling factors between samples. The absolute abundance calculations were done discretely for each tissue type, restricting opsin abundance comparisons to within-tissue.

### Fluorescent *in situ* hybridization and immunohistochemistry

Dark-adapted animals were fixed in 4% Paraformaldehyde in filtered seawater at 4 °C for 12 hours. Following fixation, they were dehydrated in a series of ethanol rinses (i.e., 25%, 50%, 75%, and 100%) then stored in 100% ethanol at −20 °C. Prior to sectioning, animals were rehydrated in ethanol/PBST (1X PBS, 0.25% tween-20) series (i.e., 75%, 50%, 25%). Whole mount pleopods photophores were obtained by sectioning 250 µm thick abdominal sections from shrimp embedded in 1.5% agarose on a Precisionary Compresstome VF-300 (Blade advance speed: 8; oscillation: 3). Whole mount maxilliped photophores were obtained by dissecting the distal segment of the 3rd maxilliped.

Based on the small size of the photophore structures and the aforementioned opsin abundance calculations, the LWS2 opsin was chosen for this analysis due to its ubiquitously high expression (see Supplementary Table 2 for expression values). pIDT blue plasmids containing 197 bp locus-specific opsin insert (i.e., LWS2) were transformed into chemically competent *Escherichia coli* and transformants were selected on LB Agar plates containing 1 mg/ml ampicillin. DNA from overnight cultures of transformed *E. coli* was isolated using QIAprep Spin Miniprep kit (Qiagen, Catalog number: 27104) following the manufacturer’s protocol. Isolated DNA was sequenced using M13 primers (forward: 5′d[GTAAAACGACGGCCAG]3′; reverse: 5′d[CAGGAAACAGCTATGAC]3′) to confirm the presence of the opsin insert. The percent identity of the 197 bp opsin insert did not exceed 52% compared to SWS opsins and we therefore expect cross-hybridization with opsin orthologs to be low.

Sense and anti-sense DIG-labeled riboprobes were prepared using template DNA from cloned bacterial colonies, DIG RNA Labeling Mix (Sigma, Catalog number: 11277073910), T7 and T3 RNA Polymerase (ThermoFisher, Catalog numbers: EP0111 and EP0101, respectively). RNA was synthesized over 4 hours at 37 °C followed by a 30-minute incubation with RNase-free DNase I (ThermoFisher, Catalog number: AM2222). Following the DNase step, free nucleotides were removed using NucAway spin columns (Invitrogen, Catalog number: AM10070). Riboprobes were visualized on a 1% DNA agarose gel and quantified using Qubit RNA BR Assay Kit (Invitrogen, Catalog number: Q10210).

Sections and whole mount tissues were treated with proteinase K (10 µg/ml) for 30 minutes. They were fixed in 4% PFA for 20 minutes to ensure the proteinase K was inhibited. Samples were pre-hybridized in hybridization solution (50% formamide, 5X SSC, 0.25% tween-20, 250 µg/ml MRE 600 tRNA, 500 µg/ml Herring Sperm DNA, pH 6.0) for 4 hours at 70 °C in a hybridization oven. The hybridization solution was replaced with fresh solution containing 1 µg/ml of riboprobes and incubated overnight at 70 °C. Remaining riboprobes were rinsed off with a series of high stringency washes (i.e., 5X SSC, 5% formamide, 0.25% tween-20; 2X SSC, 0.25% tween-20; 0.2X SSC, 0.25% tween-20) at 70 °C. Native peroxidase activity was destroyed by incubating in 2% hydrogen peroxide in PBS 0.25% tween-20 (PBST) for 1 hour. Samples were blocked using 10% Normal Goat Serum (NGS) in PBST for 4 hours. Samples were incubated at 4 °C overnight in 1:100 anti-DIG-POD Fab fragments (Sigma, Catalog number: 11207733910) in 10% NGS in PBST. Signal amplification was carried out using Tyramide conjugated to Cy5. Samples were incubated in 1:50 dilution of Tyr-Cy5 for 1 hour in the dark. Finally, samples were mounted on custom 3D printed microscope slides in Aquamount and visualized on an Olympus FV-1000 confocal microscope on an IX-81 inverted microscope.

The immunohistochemistry followed, with modification, the protocol previously reported for thick invertebrate tissue sections^61,62^ using commercially available G-alpha_q_ protein antibodies (henceforth, Gq) recently reported in scallop eyes and non-ocular photosensitive tissues^63^. The anti-Gq was designed against amino acids QLNLKEYNLV (MilliporeSigma, Catalog number: 371751), which have 100% identity with the *J. spinacauda* Gq transcript identified from RNAseq. The secondary used was goat anti-rabbit DyLight633 (Thermofisher, Catalog number: 35562). A control was run in parallel lacking primary antibodies to address possible non-specific binding of the anti-Gq antibody. Sections were first incubated in 10% hydrogen peroxide in PBS overnight to remove pigment and reduce tissue autofluorescence. Tissues were permeated with collagenase (0.5 mg/ml) and hyaluronidase (300 µg/ml) solution in PBS for 30 minutes at 37 °C then rinsed in PBS. Unspecific binding sites were blocked with 10% normal goat serum (NGS) in PBS for 2 hours. The 10% NGS solution was replaced with 1% NGS in PBS with a 1:200 dilution of the primary antibody. The primary antibody was incubated on an orbital shaker at 4 °C for 3 days. The tissues were rinsed in 10% NGS and the secondary antibody was added to 1% NGS with a dilution of 1:200. The secondary antibody was incubated on an orbital shaker at 4 °C for 3 days. Samples were rinsed in PBS and mounted on custom 3D printed microscope slides in Shield Mounting Medium with 4′,6-diamidino-2-phenylindole (DAPI) and PG (Electron Microscopy Sciences, Catalog number: 103306-190). Samples were visualized on an Olympus FV-1000 confocal microscope on an IX-81 inverted microscope.

### Experimental light exposures

For shipboard light exposure experiments, a control group remained in the dark, while two experimental groups were exposed to different levels of fluorescent room light. Room light was used to negate the possibility that any damage seen would be the result of UV wavelengths and that the light was far enough away from the holding chambers to avoid heat transfer from the light source. The “dim” group was exposed to 60 minutes of dim light at an irradiance of 1 *µ*W cm^−2^, produced by filtering room light through neutral density filtering material. This light level was the same irradiance that produced bioluminescent responses from a sergestid shrimp under laboratory conditions^64^, and should therefore be equivalent to ambient light levels experienced by these species on a daily basis. The “bright” group was exposed to 16μ*W cm*^−2^ of white light (ambient room lighting) for 60 minutes, equivalent to levels that produced ultrastructural damage in deep-sea crustacean photoreceptors^21,22,24^. After light exposure, the light was extinguished and individuals from experimental and control groups were immediately transferred under dim red light into a 2% glutaraldehyde, 0.05 M sodium cacodylate buffered seawater fixative. Pleopod photophores were dissected, prepared for transmission electron microscopy using standard techniques (stained with osmium tetroxide, dehydrated through a series of ethanol grades, infiltrated with Spurr low viscosity resin), and the ultrastructure of semithin section of the photophores was examined with a JEOL1400X TEM. Comparisons of the size/structure of the Golgi bodies and endoplasmic reticulum between control and light exposed tissue were made. Area of the basal cytoplasm (defined as any cytoplasm located between the cuticle, photocyte, nuclei and distal lateral sheath cells) and vacuoles, as well as the widths of the dorsal sheath cells was quantified using ImageJ software^65^. Normally distributed data with homogeneous variances were analyzed with ANOVAs; non-parametric data or if variances were not homogeneous were analyzed with a Kruskal-Wallis test.

## Supplementary information

Supplementary information.

## Data Availability

Raw RNA sequencing data used for these analyses are publicly available through NCBI’s Sequence Read Archive (SRA) database (BioProject ID: PRJNA521050). The tissue-specific assemblies and associated metadata are available on the Dryad Digital Repository: 10.5061/dryad.2280gb5nt.
