# Author Correction: Light organ photosensitivity in deep-sea shrimp may suggest a novel role in counterillumination

**DOI:** 10.1038/s41598-020-65514-y

**Published:** 2020-05-15

**Authors:** Heather D. Bracken-Grissom, Danielle M. DeLeo, Megan L. Porter, Tom Iwanicki, Jamie Sickles, Tamara M. Frank

**Affiliations:** 1 Department of Biology, Florida International University, North Miami, FL 33181 USA; 2 Department of Biology, University of Hawai’i at Mānoa, Honolulu, HI 96822 USA; 3 Department of Biology, Nova Southeastern University, Fort Lauderdale, FL 33314 USA

Correction to: *Scientific Reports* 10.1038/s41598-020-61284-9, published online 11 March 2020

In Figure 2, the depiction of *Janicella spinicauda* bioluminescence was an inaccurate portrayal of counterillumination in this species. The correct Figure 2 appears below as Figure 1.

**Figure 1:**
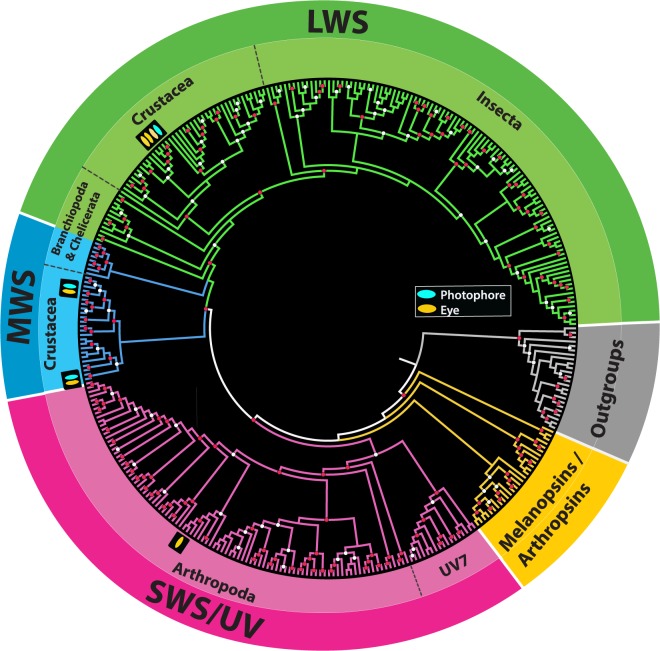
.

