# Supplementary material for: Light organ photosensitivity in deep-sea shrimp may suggest a novel role in counterillumination
Source: Sci Rep. 2020 Mar 11;10:4485. doi: 10.1038/s41598-020-61284-9 (PMC7066151; doi:10.1038/s41598-020-61284-9)
Supplement: Supplementary file 1 — Supplementary information. [file 41598_2020_61284_MOESM1_ESM.pdf]

Supplementary Material

Light organ photosensitivity in deep-sea shrimp may suggest a novel role in counterillumination

Authors: Heather D. Bracken-Grissom[1*], Danielle M. DeLeo[1], Megan L. Porter[2], Tom Iwanicki[2], Jamie Sickles[3] and Tamara M. Frank[3]

Affiliations:

[1] Department of Biology, Florida International University, North Miami, FL 33181.

[2] Department of Biology, University of Hawai'i at Mānoa, Honolulu, HI 96822.

[3] Department of Biology, Nova Southeastern University, Fort Lauderdale, FL 33314.

*Correspondence to: hbracken@fiu.edu

Supplementary Figures

Supplementary Fig. 1. Vertical phylogenetic opsin tree reconstruction comprising 325 visual rhabdomeric (r-opsins) and closely related melanopsins. Newly curated r-opsins from the *J. spinicauda* photophore (blue label) and eye (orange label) transcriptomes were aligned with a reference opsin dataset (*50, 51*) comprising visual opsins across a range of measured spectral sensitivities as well as non-visual opsins and related G-protein coupled receptors (GPCR). The putative spectral sensitivities of the r-opsins clades (SWS = short wavelength sensitive; MWS= mid-wavelength; LWS= long wavelength) were inferred from these reference datasets. The tree is comprised of two well-supported SWS clades (SWS1 and SWS2), two MWS clades (MWS1and MWS2) and two LWS clades, one specific to chelicerates- LWS1 and a second clade comprising all other putative LW opsins (LWS2). Significant triplicate bootstrap support is indicated by red circles (SH-aLRT> 80, aBayes > 0.95 and UFBoot > 95) and significant duplicate bootstrap support is indicated by white circles (SH-aLRT> 80 or UFBoot > 95, and aBayes > 0.95).

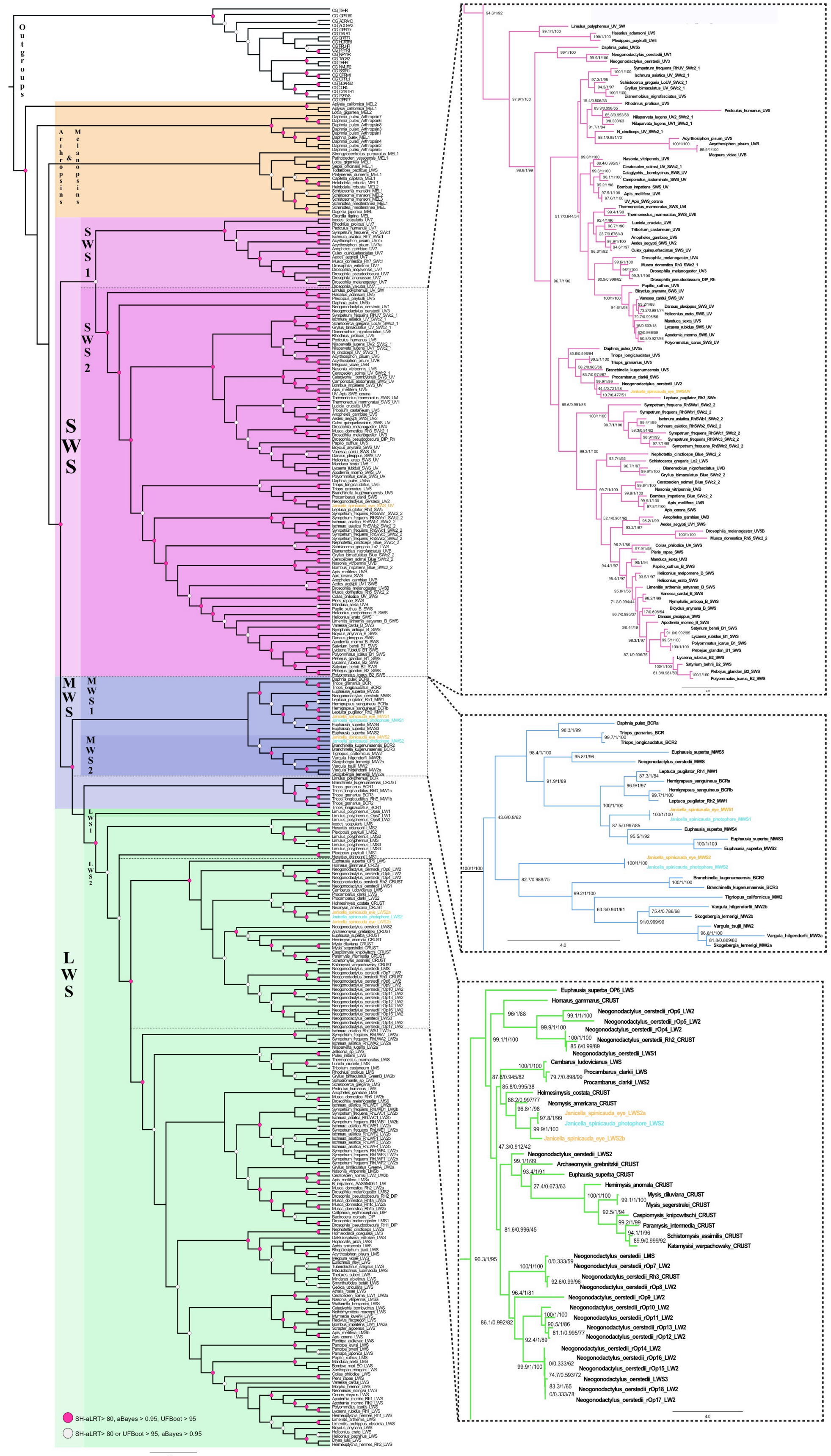

Outgroups
Arthropsins & Melanopsins
SWS
SWS1
SWS2
MWS
MWS1
MWS2
LWS
LWS1
LWS2
SH-aLRT> 80, aBayes > 0.95, UFBoot > 95
SH-aLRT> 80 or UFBoot > 95, aBayes > 0.95
4.0

Supplementary Fig. 2 - Structural alignment of *J. spinicauda* r-opsins, recovered from both the eye (E) and photophore (P) transcriptomes, against Bovine rhodopsin (2.8 Å). Putative long-wavelength sensitive (LWS) opsins are labelled in green, mid-wavelength sensitive (MWS) opsins in blue and short-wavelength sensitive, UV-shifted (SWS/UV) opsins in purple. The MWS opsin from the crab *Hemigrapsus sanguineus* (BCRa) was included for comparison. This opsin has an experimentally determined spectral sensitivity (λ_{max}) of 480 nm (Sakamoto et al. 1996) and appears closely related to the *J. spinicauda* MWS1 opsins. Alpha (α) helices represent the seven transmembrane domains characteristic of (Bovine) rhodopsin. Bolded residues, shaded gray represent the following conserved domains, motifs and residues (numbered in blue), which are characteristic of invertebrate r-opsins (as described in Katti et al. 2010): (1) two conserved cysteine (C) residues, (2) the site equivalent to *Bovine G90* where lysine (K) indicates UV sensitivity in invertebrates (Salcedo et al. 2003), (3) the site equivalent to the Schiff base counterion (*Bovine E113*) which contributes sensitivity between visible light (Y: Tyrosine) and UV light (F: phenylalanine), (4) the (E/D)RY motif conserved in G-protein coupled receptors at the cytoplasmic end of helix III, (5) the "R(E/D)QAKKMN" sequence conserved among arthropod opsins, (6) a conserved Lys residue critical for Schiff base formation with the chromophore and (7) an amino acid triplet (HP(R/K)) known to couple to Gq-alpha. Amino acid residues in red represent similar residues within a given column (based on the calculated percentage of equivalent residues in terms of physico-chemical properties).

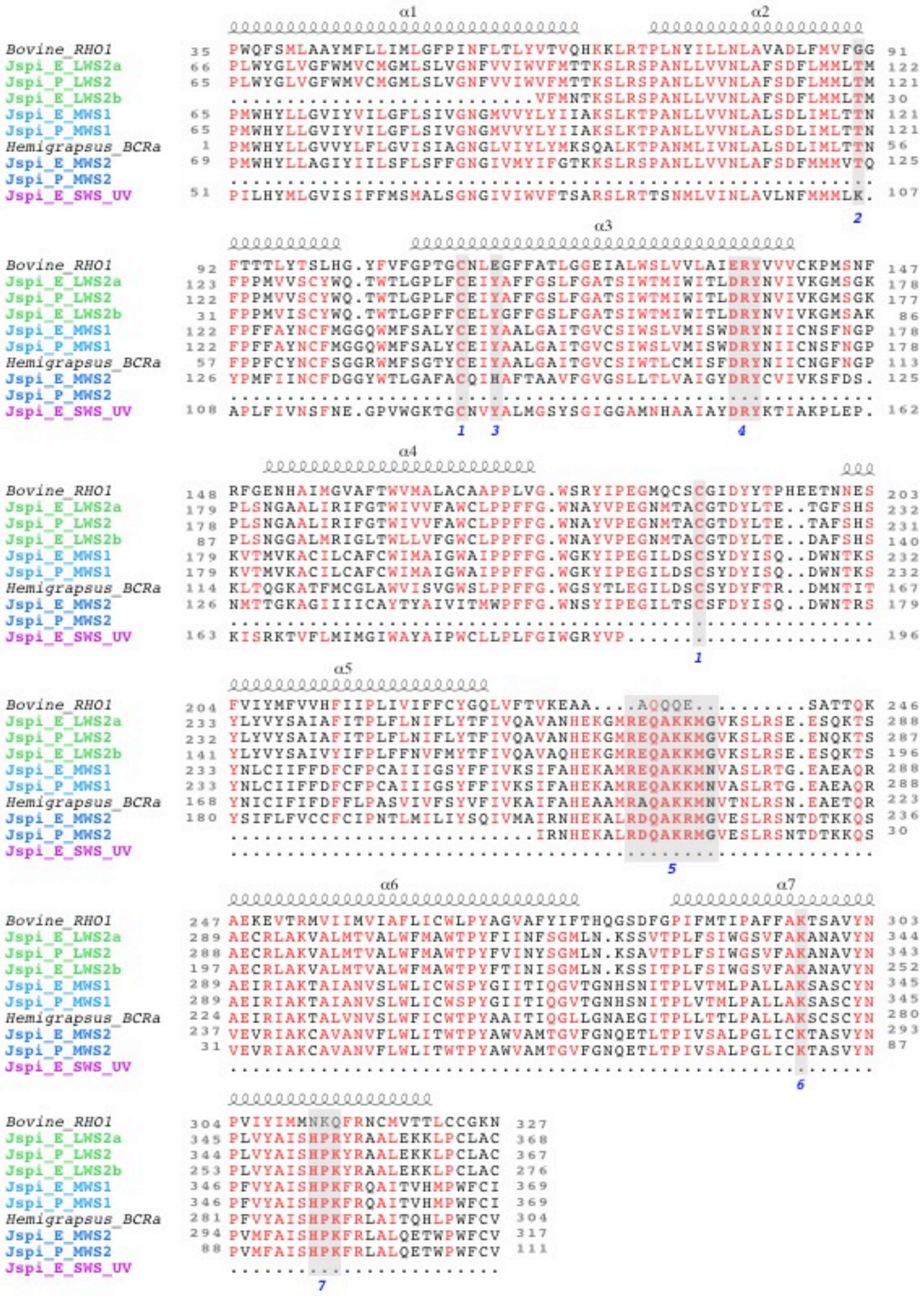

α1
α2
α3
α4
α5
α6
α7
Bovine_RHO1
Jspi_E_LWS2a
Jspi_P_LWS2
Jspi_E_LWS2b
Jspi_E_MWS1
Jspi_P_MWS1
Hemigrapsus_BCRa
Jspi_E_MWS2
Jspi_P_MWS2
Jspi_E_SWS_UV

Supplementary Fig. 3. Examples of *in situ* hybridization sense riboprobes control and immunohistochemistry no-primary controls in maxilliped (whole mount) and pleopod (100 µm thick sections) photophores of *J. spinicauda*. (A) LWS sense riboprobes exhibit low background and no visible labeling in maxilliped photophores. (B, C) No primary control for Gq protein assay exhibit virtually no background or labeling in the apex of photophores (as in Fig. 3). Gq protein assay (magenta) and LWS sense riboprobes (red) are labeled with Cy5; cell nuclei (green) are labeled with diamidino-2-phenylidole (DAPI); tissue autofluorescence is also present (diffuse red and green). Single arrowheads point to apex of individual photocytes containing paracrystalline bodies and double arrowheads point to photocyte nuclei in the distal region of photophores. Individual photocytes are outlined with dashed white line (A,B,C). (di distal, do dorsal, la lateral, c cuticle). Note: Dorsal oriented downward. Refer to Fig 1. for photophore structure and light microscopy images.

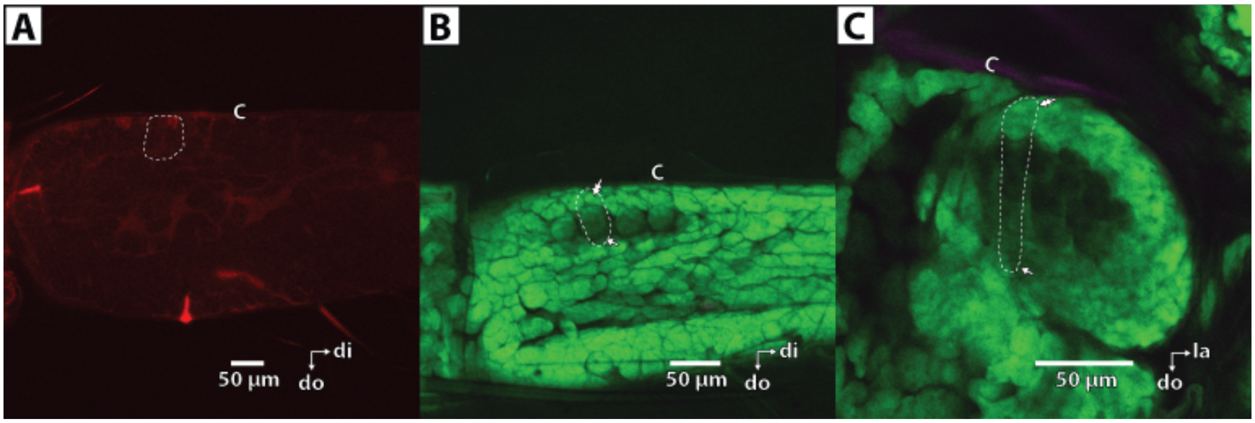

Supplementary Tables

Supplementary Table 1. Assembly statistics for tissue-specific (eye and photophore) transcriptomes of *Janicella spinicauda.*

Janicella spinicauda **tissue specific reference assemblies**

Metric	Eyes	Photophores
Number of transcripts	140,357	201,159
Mean transcript length (bp)	616.5	781.9
Reconstruction size (bases)	86,532,701	157,279,829
Transcripts over 1K bp	17,139	42,682
Transcripts over 10K bp	15	8
Number of contigs with ORFs	20,304	43,940
Mean ORF percent	50.6	52.8
GC content (%)	37.2	40.2
N50 (longest isoform)	908	1356
N50 (all transcripts)	747	1232

Supplementary Table 2. TMM (Trimmed Mean of *M-values*) normalized opsin abundance counts for each tissue type, eye and photophore. The TMM-normalization method uses a weighted trimmed mean of the log expression ratios to estimate scaling factors between samples (Robinson et al. 2010). Tissue-specific TMM expression was calculated from transcript per million (TPM) abundance estimates generated with Salmon (Patro et al. 2017) in order to compare absolute abundance measures between opsin clades. Note- absolute abundance comparisons are restricted to tissue type.

Tissue	*Opsin clade*	*Transcript ID*	*Average TMM expression*
Eyes	SWS/UV	TRINITY_DN16415_c0_g1	1.6
	MWS1	TRINITY_DN7379_c0_g1	20.9
	MWS2	TRINITY_DN4693_c0_g1	1157.9
	LWS2a	TRINITY_DN11104_c0_g1	8068.4
	LWS2b	TRINITY_DN7709_c0_g1_ext	5110.7
Photophores	MWS1	TRINITY_DN45546_c0_g1	1.1
	MWS2	TRINITY_DN31556_c0_g1	2.0
	LWS2	TRINITY_DN14462_c0_g1	12.4

Supplementary Table 3. Shipboard light-exposure experiment statistics. *Janicella spinacauda* 60-minute control (dark), dim and bright light-exposed tissue.

**Photophore Organelle**	**Light Level**<br>**Dark (n=6)**	**Dim (n=6)**	**Test/*p* value**
	**µm ± S.E.**	**µm ± S.E.**	
Basal Cytoplasm Area	296.62 ± 47.91	217.05 ± 72.30	One-Way ANOVA, *p* =0.48
Vacuole Area	114.67 ± 7.10	51.07 ± 18.21	One-Way ANOVA, *p* =0.04
Sheath Cell Width	0.95 ± 0.04	1.20 ± 0.04	Kruskal-Wallis, *p* <0.01

**Photophore Organelle**	**Light Level**<br>**Dark (n=6)**	**Bright (n=7)**	**Test/*p* value**
	**µm ± S.E.**	**µm ± S.E.**	
Basal Cytoplasm Area	296.62 ± 47.91	1483.9 ± 351.65	One-Way ANOVA, *p* =0
Vacuole Area	114.67 ± 7.10	749.00 ± 277.76	One-Way ANOVA, *p* =0
Sheath Cell Width	0.95 ± 0.04	2.17 ± 0.08	Kruskal-Wallis, p <0.01

Supplementary Table 4. Opsin metadata table with species information, accession number and inferred opsin identity.

Class	Order	Family	*Species*	Common name	Accession ID	Opsin ID
Arachnida	Araneae	Salticidae	*Hasarius adansoni*	jumping spider	BAG14330	LMS1
Arachnida	Araneae	Salticidae	*Hasarius adansoni*	jumping spider	BAG14331	LMS2
Arachnida	Araneae	Salticidae	*Hasarius adansoni*	jumping spider	BAG14332	UV5
Arachnida	Ixodida	Ixodidae	*Ixodes scapularis*	deer tick	XM_002408275.1	LMS
Arachnida	Ixodida	Ixodidae	*Ixodes scapularis*	deer tick	Genome	UV7
Arachnida	Araneae	Salticidae	*Plexippus paykulli*	jumping spider	BAG14333.1	LMS1
Arachnida	Araneae	Salticidae	*Plexippus paykulli*	jumping spider	BAG14334.1	LMS2
Arachnida	Araneae	Salticidae	*Plexippus paykulli*	jumping spider	BAG14335	UV5
Branchiopoda	Anostraca	Thamnocephalidae	*Branchinella kugenumaensis*	fairy shrimp	BAG80986	RhD/BCR2
Branchiopoda	Anostraca	Thamnocephalidae	*Branchinella kugenumaensis*	fairy shrimp	BAG80985	RhC/BCR3
Branchiopoda	Anostraca	Thamnocephalidae	*Branchinella kugenumaensis*	fairy shrimp	BAG80987	RhB/CRUST
Branchiopoda	Anostraca	Thamnocephalidae	*Branchinella kugenumaensis*	fairy shrimp	BAG80984	RhA/UV5
Branchiopoda	Diplostraca	Daphniidae	*Daphnia pulex*	water flea	Genome	Arthropsin1
Branchiopoda	Diplostraca	Daphniidae	*Daphnia pulex*	water flea	Genome	Arthropsin2
Branchiopoda	Diplostraca	Daphniidae	*Daphnia pulex*	water flea	Genome	Arthropsin3
Branchiopoda	Diplostraca	Daphniidae	*Daphnia pulex*	water flea	Genome	Arthropsin4
Branchiopoda	Diplostraca	Daphniidae	*Daphnia pulex*	water flea	Genome	Arthropsin5
Branchiopoda	Diplostraca	Daphniidae	*Daphnia pulex*	water flea	Genome	Arthropsin6
Branchiopoda	Diplostraca	Daphniidae	*Daphnia pulex*	water flea	Genome	Arthropsin7
Branchiopoda	Diplostraca	Daphniidae	*Daphnia pulex*	water flea	Genome	Arthropsin8
Branchiopoda	Diplostraca	Daphniidae	*Daphnia pulex*	water flea	Genome	BCRa
Branchiopoda	Diplostraca	Daphniidae	*Daphnia pulex*	water flea	EFX75461	Blue/UV5a
Branchiopoda	Diplostraca	Daphniidae	*Daphnia pulex*	water flea	EFX81332	UV5b
Branchiopoda	Diplostraca	Daphniidae	*Daphnia pulex*	water flea	Genome	MEL1
Branchiopoda	Notostraca	Triopsidae	*Triops granarius*	desert tadepole shrimp	BAG80976	RhA/BCR
Branchiopoda	Notostraca	Triopsidae	*Triops granarius*	desert tadepole shrimp	BAG80979	RhD/BCR1
Branchiopoda	Notostraca	Triopsidae	*Triops granarius*	desert tadepole shrimp	BAG80977	RhB/BCR2
Branchiopoda	Notostraca	Triopsidae	*Triops granarius*	desert tadepole shrimp	BAG80980	RhE/BCR3
Branchiopoda	Notostraca	Triopsidae	*Triops granarius*	desert tadepole shrimp	BAG80978	RhC/UV5
Branchiopoda	Notostraca	Triopsidae	*Triops longicaudatus*	tadepole shrimp	BAG80982	RhB/BCR1
Branchiopoda	Notostraca	Triopsidae	*Triops longicaudatus*	tadepole shrimp	BAG80981	RhABCR2
Branchiopoda	Notostraca	Triopsidae	*Triops longicaudatus*	tadepole shrimp	BAG80983	RhC/UV6

Branchiopoda	Notostraca	Triopsidae	*Triops longicaudatus*	tadepole shrimp	BAG80998	RhD/MW1
Branchiopoda	Notostraca	Triopsidae	*Triops longicaudatus*	tadepole shrimp	BAG80999	RhE/MW1
Copepoda	Harpacticoida	Harpacticidae	*Tigriopus californicus*	tidepool copepod	ADZ45237	MW2
Insecta	Hemiptera	Aphididae	*Acyrthosiphon pisum*	pea aphid	XP_001947730	LMS
Insecta	Hemiptera	Aphididae	*Acyrthosiphon pisum*	pea aphid	187892212	LWS
Insecta	Hemiptera	Aphididae	*Acyrthosiphon pisum*	pea aphid	XP_001951588	UV5
Insecta	Hemiptera	Aphididae	*Acyrthosiphon pisum*	pea aphid	XP_001950416	UV/Rh7a
Insecta	Hemiptera	Aphididae	*Acyrthosiphon pisum*	pea aphid	XP_001944926	UV/Rh7b
Insecta	Diptera	Culicidae	*Aedes aegypti*	yellow fever mosquito	XP_001662982	SWS/UV1
Insecta	Diptera	Culicidae	*Aedes aegypti*	yellow fever mosquito	XP_001653866	SWS/UV2
Insecta	Diptera	Culicidae	*Aedes aegypti*	yellow fever mosquito	XM_001650694	UV/RH7
Insecta	Diptera	Culicidae	*Anopheles gambiae*	African malaria mosquito	O97165	LMS
Insecta	Diptera	Culicidae	*Anopheles gambiae*	African malaria mosquito	XP_001688790	UV5
Insecta	Diptera	Culicidae	*Anopheles gambiae*	African malaria mosquito	XP_308329	UV7
Insecta	Diptera	Culicidae	*Anopheles gambiae*	African malaria mosquito	XP_319247	UVB
Insecta	Hemiptera	Aphididae	*Aphis spiraecola*	spirea aphid	FM177115	LWS
Insecta	Hymenoptera	Apidae	*Apis cerana*	Eastern honey bee	219566008	LWS
Insecta	Hymenoptera	Apidae	*Apis cerana*	Eastern honey bee	BAH04515.1	SWS
Insecta	Hymenoptera	Apidae	*Apis cerana*	Eastern honey bee	BAH04514.1	SWS
Insecta	Hymenoptera	Apidae	*Apis mellifera*	honey bee	AAA69069	LWS1
Insecta	Hymenoptera	Apidae	*Apis mellifera*	honey bee	DAA05740	LWS2
Insecta	Hymenoptera	Apidae	*Apis mellifera*	honey bee	AAC13418	UV5
Insecta	Hymenoptera	Apidae	*Apis mellifera*	honey bee	AAC13417	Blue
Insecta	Lepidoptera	Riodinidae	*Apodemia mormo*	Mormon metalmark butterfly	50981744	Rh1/LWS
Insecta	Lepidoptera	Riodinidae	*Apodemia mormo*	Mormon metalmark butterfly	50981746	Rh2/LWS
Insecta	Lepidoptera	Riodinidae	*Apodemia mormo*	Mormon metalmark butterfly	AAT91643.1	SWS
Insecta	Lepidoptera	Riodinidae	*Apodemia mormo*	Mormon metalmark butterfly	AAT91642.1	SWS
Insecta	Hymenoptera	Tenthredinidae	*Athalia rosae*	turnip sawfly	188593534	LWS
Insecta	Diptera	Tephritidae	*Bactrocera dorsalis*	Oriental fruit fly	AAS88872	DIP/Rh1
Insecta	Lepidoptera	Nymphalidae	*Bicyclus anynana*	squinting bush brown butterfly	157502893	LWS
Insecta	Lepidoptera	Nymphalidae	*Bicyclus anynana*	squinting bush brown butterfly	AAY16527.1	SWS
Insecta	Lepidoptera	Nymphalidae	*Bicyclus anynana*	squinting bush brown butterfly	AAL91507.1	SWS

Insecta	Lepidoptera	Apidae	*Bombus impatiens*	common eastern bumblebee	AAV67326	UV
Insecta	Hymenoptera	Apidae	*Bombus impatiens*	common eastern bumblebee	AAS55402	LW1
Insecta	Hymenoptera	Apidae	*Bombus impatiens*	common eastern bumblebee	AAS55406	LW2
Insecta	Hymenoptera	Apidae	*Bombus impatiens*	common eastern bumblebee	XP_003494923	Blue
Insecta	Lepidoptera	Bombycidae	*Bombyx mori*	domestic silkmoth	112983528	LWS
Insecta	Diptera	Calliphoridae	*Calliphora vicina (erythrocephala)*	urban bluebottle blowfly	156511	DIP/opsin
Insecta	Hymenoptera	Formicidae	*Camponotus abdominalis*	ant	AAC05092.1	SWS
Insecta	Hymenoptera	Formicidae	*Cataglyphis bombycinus*	saharan silver ant	AF042787.1	LWS
Insecta	Hymenoptera	Formicidae	*Cataglyphis bombycinus*	saharan silver ant	AAC05091.1	SWS
Insecta	Hymenoptera	Agaonidae	*Ceratosolen solmsi*	fig wasp	AGH15790	LW1
Insecta	Hymenoptera	Agaonidae	*Ceratosolen solmsi*	fig wasp	AGH15791	LW2
Insecta	Hymenoptera	Agaonidae	*Ceratosolen solmsi*	fig wasp	AGH15792	Blue
Insecta	Hymenoptera	Agaonidae	*Ceratosolen solmsi*	fig wasp	AGH15793	UV
Insecta	Lepidoptera	Pieridae	*Colias philodice*	clouded sulfer north american butterfly	62860651	LWS
Insecta	Lepidoptera	Pieridae	*Colias philodice*	clouded sulfer north american butterfly	AAY16532.1	SWS
Insecta	Diptera	Culicidae	*Culex quinquefasciatus*	southern house mosquito	XP_001851157	SWS
Insecta	Diptera	Culicidae	*Culex quinquefasciatus*	southern house mosquito	XM_001861603	UV7
Insecta	Hemiptera	Phylloxeridae	*Daktulosphaira vitifoliae*	grape phylloxera	22796138	LWS
Insecta	Lepidoptera	Nymphalidae	*Danaus plexippus*	monarch butterfly	AAU07977.1	SWS
Insecta	Lepidoptera	Nymphalidae	*Danaus plexippus*	monarch butterfly	AAU07979.1	SWS
Insecta	Orthoptera	Trigonidiidae	*Dianemobius nigrofasciatus*	band-legged ground cricket	BAG71429	UV5
Insecta	Orthoptera	Trigonidiidae	*Dianemobius nigrofasciatus*	band-legged ground cricket	BAF45422	UVB
Insecta	Diptera	Drosophilidae	*Drosophila ananassae*	fruit fly	XP_001956024	UV/RH7
Insecta	Diptera	Drosophilidae	*Drosophila melanogaster*	fruit fly	AAA28733	Rh1/LW
Insecta	Diptera	Drosophilidae	*Drosophila melanogaster*	fruit fly	AAA28734	Rh2/LW
Insecta	Diptera	Drosophilidae	*Drosophila melanogaster*	fruit fly	CAB06821	Rh6/LW
Insecta	Diptera	Drosophilidae	*Drosophila melanogaster*	fruit fly	AAA28854	RH3/UV3
Insecta	Diptera	Drosophilidae	*Drosophila melanogaster*	fruit fly	AAA28856	UV/RH4
Insecta	Diptera	Drosophilidae	*Drosophila melanogaster*	fruit fly	AAC47426	Rh5/Blue/UV5B
Insecta	Diptera	Drosophilidae	*Drosophila melanogaster*	fruit fly	NP_524035	UV/RH7
Insecta	Diptera	Drosophilidae	*Drosophila mojavensis*	fruit fly	XP_002007363.1	UV/RH7
Insecta	Diptera	Drosophilidae	*Drosophila pseudoobscura*	fruit fly	9077	RH1/DIP

Insecta	Diptera	Drosophilidae	*Drosophila pseudoobscura*	fruit fly	9079	RH2/DIP
Insecta	Diptera	Drosophilidae	*Drosophila pseudoobscura*	fruit fly	CAA46710	RH3/DIP
Insecta	Diptera	Drosophilidae	*Drosophila pseudoobscura*	fruit fly	XP_002134833	UV/RH7
Insecta	Diptera	Drosophilidae	*Drosophila willistoni*	fruit fly	XP_002068301.1	UV/RH7
Insecta	Diptera	Drosophilidae	*Drosophila yakuba*	fruit fly	XP_002094554	UV/RH7
Insecta	Lepidoptera	Nymphalidae	*Dryas iulia*	Julia butterfly	14276143	LWS
Insecta	Hemiptera	Aphididae	*Eulachnus rileyi*	pine needle aphid	22796134	LWS
Insecta	Hemiptera	Aphididae	*Geoica utricularia*	aphid	219920848	LWS
Insecta	Orthoptera	Gryllidae	*Gryllus bimaculatus*	two-spotted cricket	AEG78683	GreenA/LW
Insecta	Orthoptera	Gryllidae	*Gryllus bimaculatus*	two-spotted cricket	AEG78684	GreenB/LW
Insecta	Orthoptera	Gryllidae	*Gryllus bimaculatus*	two-spotted cricket	AEG78685	Blue/SW
Insecta	Orthoptera	Gryllidae	*Gryllus bimaculatus*	two-spotted cricket	AEG78686	UV
Insecta	Lepidoptera	Nymphalidae	*Heliconius erato*	red postman butterfly	62860665	LWS
Insecta	Lepidoptera	Nymphalidae	*Heliconius erato*	red postman butterfly	AAY16539.1	SWS
Insecta	Lepidoptera	Nymphalidae	*Heliconius erato*	red postman butterfly	AAY16537.1	SWS
Insecta	Lepidoptera	Nymphalidae	*Heliconius melpomene*	postman butterfly	AAY16530.1	SWS
Insecta	Lepidoptera	Nymphalidae	*Heliconius pachinus*	longwing butterfly	14276141	LWS
Insecta	Lepidoptera	Nymphalidae	*Hermeuptychia hermes*	hermes satyr butterfly	ABI97874.1	Rh1/LWS
Insecta	Lepidoptera	Nymphalidae	*Hermeuptychia hermes*	hermes satyr butterfly	115490645	Rh2/LWS
Insecta	Hemiptera	Cicadellidae	*Homalodisca coagulata*	glassy-winged sharpshooter	AY588065	LMS
Insecta	Hemiptera	Aphididae	*Hoplocallis picta*	painted holm oak aphid	31442124	LWS
Insecta	Odonata	Coenagrionidae	*Ischnura asiatica*	damselfly	BAQ54909	RhLWA1
Insecta	Odonata	Coenagrionidae	*Ischnura asiatica*	damselfly	BAQ54910	RhLWA2
Insecta	Odonata	Coenagrionidae	*Ischnura asiatica*	damselfly	BAQ54911	RhLWC1
Insecta	Odonata	Coenagrionidae	*Ischnura asiatica*	damselfly	BAQ54912	RhLWD1
Insecta	Odonata	Coenagrionidae	*Ischnura asiatica*	damselfly	BAQ54913	RhLWE1
Insecta	Odonata	Coenagrionidae	*Ischnura asiatica*	damselfly	BAQ54914	RhLWF1
Insecta	Odonata	Coenagrionidae	*Ischnura asiatica*	damselfly	BAQ54915	RhLWF2
Insecta	Odonata	Coenagrionidae	*Ischnura asiatica*	damselfly	BAQ54916	RhLWF3
Insecta	Odonata	Coenagrionidae	*Ischnura asiatica*	damselfly	BAQ54917	RhLWF4
Insecta	Odonata	Coenagrionidae	*Ischnura asiatica*	damselfly	BAQ54918	RhSWb1
Insecta	Odonata	Coenagrionidae	*Ischnura asiatica*	damselfly	BAQ54919	RhSWb2
Insecta	Odonata	Coenagrionidae	*Ischnura asiatica*	damselfly	BAQ54920	RhUV

Insecta	Odonata	Coenagrionidae	*Ischnura asiatica*	damselfly	BAQ54908	UV/Rh7
Insecta	Siphonaptera	Ceratophyllidae	*Jellisonia sp*	flea	58615727	LWS
Insecta	Lepidoptera	Nymphalidae	*Limenitis archippus obsoleta*	viceroy butterfly	ABA44351.1	LWS
Insecta	Lepidoptera	Nymphalidae	*Limenitis arthemis*	admiral butterfly	62860657	LWS
Insecta	Lepidoptera	Nymphalidae	*Limenitis arthemis astyanax*	admiral butterfly	AAY16535.2	SWS
Insecta	Coleoptera	Lampyridae	*Luciola cruciata*	japanese firefly	BAH56227.1	LMS
Insecta	Coleoptera	Lampyridae	*Luciola cruciata*	japanese firefly	BAH56228	UV5
Insecta	Lepidoptera	Nymphalidae	*Lycaena rubidus*	ruddy copper butterfly	50981732	Rh1/LWS
Insecta	Lepidoptera	Nymphalidae	*Lycaena rubidus*	ruddy copper butterfly	AAT91639.1	B1/SWS
Insecta	Lepidoptera	Nymphalidae	*Lycaena rubidus*	ruddy copper butterfly	AAT91640.1	B2/SWS
Insecta	Lepidoptera	Nymphalidae	*Lycaena rubidus*	ruddy copper butterfly	AAT91641.1	SWS
Insecta	Lepidoptera	Nymphalidae	*Maculolachnus submacula*	ruddy copper butterfly	219920834	LWS
Insecta	Lepidoptera	Sphingidae	*Manduca sexta*	tobacco hornworm	O02464	ManOp1/LMS
Insecta	Lepidoptera	Sphingidae	*Manduca sexta*	tobacco hornworm	O02465	Manop2/UV5
Insecta	Lepidoptera	Sphingidae	*Manduca sexta*	tobacco hornworm	O96107	Manop3/UVB
Insecta	Hemiptera	Aphididae	*Megoura viciae*	green aphid	AF189714	LWS
Insecta	Hemiptera	Aphididae	*Megoura viciae*	green aphid	AAG17120	UVB
Insecta	Hemiptera	Aphididae	*Mindarus abietinus*	balsam twig aphid	219920842	LWS
Insecta	Hemiptera	Aphididae	*Morpho helenor*	balsam twig aphid	115490633	LWS
Insecta	Diptera	Muscidae	*Musca domestica*	house fly	XP_005182995	Rh1a /LW2a
Insecta	Diptera	Muscidae	*Musca domestica*	house fly	XP_011291215	Rh1b /LW2a
Insecta	Diptera	Muscidae	*Musca domestica*	house fly	XP_005182983	Rh1c /LW2a
Insecta	Diptera	Muscidae	*Musca domestica*	house fly	XP_005191160	Rh2 /LW2a
Insecta	Diptera	Muscidae	*Musca domestica*	house fly	XP_005186097	Rh6 /LW2b
Insecta	Diptera	Muscidae	*Musca domestica*	house fly	XP_005175090	Rh5/ SWc2.2
Insecta	Diptera	Muscidae	*Musca domestica*	house fly	XP_005190072	Rh3/ SWc2.1
Insecta	Diptera	Muscidae	*Musca domestica*	house fly	XP_005181535	UV/Rh7 /SWc1
Insecta	Hymenoptera	Formicidae	*Myrmecia loweryi*	giant bull ant	75755591	LWS
Insecta	Hymenoptera	Pteromalidae	*Nasonia vitripennis*	parasitoid wasp	NM_001170908.1	LMSa
Insecta	Hymenoptera	Pteromalidae	*Nasonia vitripennis*	parasitoid wasp	NM_001170908.1	LMSb
Insecta	Hymenoptera	Pteromalidae	*Nasonia vitripennis*	parasitoid wasp	XP_001608074	UV5
Insecta	Hymenoptera	Pteromalidae	*Nasonia vitripennis*	parasitoid wasp	XP_001604622.1	UVB
Insecta	Lepidoptera	Nymphalidae	*Neominois ridingsii*	ridings' satyr butterfly	115490647	LWS

Insecta	Hemiptera	Cicadellidae	*Nephotettix cincticeps*	green rice leafhopper	BAO03864	LW
Insecta	Hemiptera	Cicadellidae	*Nephotettix cincticeps*	green rice leafhopper	BAO03865	Blue
Insecta	Hemiptera	Cicadellidae	*Nephotettix cincticeps*	green rice leafhopper	BAO03866	UV
Insecta	Hemiptera	Delphacidae	*Nilaparvata lugens*	brown planthopper	BAO03855	LW2a
Insecta	Hemiptera	Delphacidae	*Nilaparvata lugens*	brown planthopper	BAO03856	UV1
Insecta	Hemiptera	Delphacidae	*Nilaparvata lugens*	brown planthopper	BAO03857	UV2
Insecta	Hymenoptera	Formicidae	*Nothomyrmecia macrops*	dinosaur (dawn) ant	75755597	LWS
Insecta	Lepidoptera	Nymphalidae	*Nymphalis antiopa*	mourning cloak butterfly	AAY16526.1	SWS
Insecta	Lepidoptera	Nymphalidae	*Oeneis chryxus*	brown artic butterfly	115490649	LWS
Insecta	Mecoptera	Panorpidae	*Panorpa arakavae*	scorpin fly	58615717	LWS
Insecta	Mecoptera	Panorpidae	*Panorpa japonica*	scorpin fly	58615719	LWS
Insecta	Mecoptera	Panorpidae	*Panorpa lewisi*	scorpin fly	58615721	LWS
Insecta	Mecoptera	Panorpidae	*Panorpa pryeri*	scorpin fly	58615715	LWS
Insecta	Lepidoptera	Papilionidae	*Papilio xuthus*	asian swallowtail butterfly	AB028218	LMS
Insecta	Lepidoptera	Papilionidae	*Papilio xuthus*	asian swallowtail butterfly	BAA93469.1	SWS
Insecta	Lepidoptera	Papilionidae	*Papilio xuthus*	asian swallowtail butterfly	BAA93470	UV5
Insecta	Phthiraptera (Psocodea)	Pediculidae	*Pediculus humanus*	human body louse	XP_002427337	LWS
Insecta	Phthiraptera (Psocodea)	Pediculidae	*Pediculus humanus*	human body louse	XP_002422743	Op2/UV
Insecta	Phthiraptera (Psocodea)	Pediculidae	*Pediculus humanus*	human body louse	XP_002432663	UV/RH7
Insecta	Lepidoptera	Pieridae	*Pieris rapae*	cabbage white butterfly	BAD06459	LWS
Insecta	Lepidoptera	Pieridae	*Pieris rapae*	cabbage white butterfly	BAE19945.1	SWS
Insecta	Lepidoptera	Lycaenidae	*Plebejus glandon*	arctic blue butterfly	ABD64152.1	B1/SWS
Insecta	Lepidoptera	Lycaenidae	*Plebejus glandon*	arctic blue butterfly	ABD64153.1	B2/SWS
Insecta	Lepidoptera	Lycaenidae	*Polyommatus icarus*	common blue butterfly	158512151	LWS
Insecta	Lepidoptera	Lycaenidae	*Polyommatus icarus*	common blue butterfly	ABD64150.1	B1/SWS
Insecta	Lepidoptera	Lycaenidae	*Polyommatus icarus*	common blue butterfly	ABD64151.1	B2/SWS
Insecta	Lepidoptera	Lycaenidae	*Polyommatus icarus*	common blue butterfly	ABW69111.1	SWS
Insecta	Siphonaptera	Pulicidae	*Pulex irritans*	human flea	58615723	LWS
Insecta	Hymenoptera	Melittidae	*Rediviva mcgregori*	bee	AAZ94257	LWS
Insecta	Hemiptera	Reduviidae	*Rhodnius prolixus*	kissing bug	Genome	LMS
Insecta	Hemiptera	Reduviidae	*Rhodnius prolixus*	kissing bug	Genome	UV5
Insecta	Hemiptera	Reduviidae	*Rhodnius prolixus*	kissing bug	Genome	UV7
Insecta	Hemiptera	Aphididae	*Rhopalosiphum padi*	bird cherry-oat aphid	FM177114	LWS

Insecta	Lepidoptera	Lycaenidae	*Satyrium behrii*	Behr's hairstreak butterfly	ABD64148.1	B1/SWS
Insecta	Lepidoptera	Lycaenidae	*Satyrium behrii*	Behr's hairstreak butterfly	ABD64149.1	B2/SWS
Insecta	Orthoptera	Acrididae	*Schistocerca gregaria*	desert locust	BAP16681	LoUV
Insecta	Orthoptera	Acrididae	*Schistocerca gregaria*	desert locust	CAA56377	Lo1/LW
Insecta	Orthoptera	Acrididae	*Schistocerca gregaria*	desert locust	CAA56378	Lo2/Blue
Insecta	Hymenoptera	Colletidae	*Scrapter algoensis*	bee	ABM92898	LWS
Insecta	Hemiptera	Aphididae	*Smynthurodes betae*	bean root aphid	219920850	LWS
Insecta	Mantodea	Mantidae	*Sphodromantis sp*	praying mantis	312514	LWS
Insecta	Odonata	Libellulidae	*Sympetrum frequens*	red dragonfly	BAQ54698	RhLWA1
Insecta	Odonata	Libellulidae	*Sympetrum frequens*	red dragonfly	BAQ54699	RhLWA2
Insecta	Odonata	Libellulidae	*Sympetrum frequens*	red dragonfly	BAQ54700	RhLWB1
Insecta	Odonata	Libellulidae	*Sympetrum frequens*	red dragonfly	BAQ54701	RhLWC1
Insecta	Odonata	Libellulidae	*Sympetrum frequens*	red dragonfly	BAQ54702	RhLWD1
Insecta	Odonata	Libellulidae	*Sympetrum frequens*	red dragonfly	BAQ54703	RhLWE1
Insecta	Odonata	Libellulidae	*Sympetrum frequens*	red dragonfly	BAQ54704	RhLWF1
Insecta	Odonata	Libellulidae	*Sympetrum frequens*	red dragonfly	BAQ54705	RhLWF2
Insecta	Odonata	Libellulidae	*Sympetrum frequens*	red dragonfly	BAQ54706	RhLWF3
Insecta	Odonata	Libellulidae	*Sympetrum frequens*	red dragonfly	BAQ54707	RhLWF4
Insecta	Odonata	Libellulidae	*Sympetrum frequens*	red dragonfly	BAQ54708	RhSWa1
Insecta	Odonata	Libellulidae	*Sympetrum frequens*	red dragonfly	BAQ54709	RhSWb1
Insecta	Odonata	Libellulidae	*Sympetrum frequens*	red dragonfly	BAQ54710	RhSWc1
Insecta	Odonata	Libellulidae	*Sympetrum frequens*	red dragonfly	BAQ54711	RhSWc2
Insecta	Odonata	Libellulidae	*Sympetrum frequens*	red dragonfly	BAQ54712	RhSWc3
Insecta	Odonata	Libellulidae	*Sympetrum frequens*	red dragonfly	BAQ54713	RhUV
Insecta	Odonata	Libellulidae	*Sympetrum frequens*	red dragonfly	BAQ54697	UV/Rh7
Insecta	Hemiptera	Aphididae	*Thelaxes suberi*	Southern oak thelaxid	22797870	LWS
Insecta	Coleoptera	Dytiscidae	*Thermonectus marmoratus*	Sunburst diving beetle	197259972	LWS
Insecta	Coleoptera	Dytiscidae	*Thermonectus marmoratus*	Sunburst diving beetle	ACH56537.1	SWS
Insecta	Coleoptera	Dytiscidae	*Thermonectus marmoratus*	Sunburst diving beetle	ACH56538.1	SWS
Insecta	Coleoptera	Tenebrionidae	*Tribolium castaneum*	red flour beetle	NP_001155991	LMS/LW
Insecta	Coleoptera	Tenebrionidae	*Tribolium castaneum*	red flour beetle	XP_970344	UV5
Insecta	Hemiptera	Aphididae	*Tuberolachnus salignus*	giant willow aphid	FM177113	LWS
Insecta	Lepidoptera	Nymphalidae	*Vanessa cardui*	painted lady butterfly	159798088	LWS

Insecta	Lepidoptera	Nymphalidae	*Vanessa cardui*	painted lady butterfly	AAP49026.2	SWS
Insecta	Lepidoptera	Nymphalidae	*Vanessa cardui*	painted lady butterfly	AAP49025.2	SWS
Insecta	Hymenoptera	Agaonidae	*Walkerella benjamini*	fig wasp	157326182	LWS
Insecta	Lepidoptera	Sphingidae	*Xanthopan morgani*	Morgan's sphinx moth	37719669	LWS
Malacostraca	Mysida	Mysidae	*Archaeomysis grebnitzkii*	-	DQ852575	CRUST
Malacostraca	Decapoda	Cambaridae	*Cambarus ludovicianus*	painted devil crayfish	CK991518.1	LWS
Malacostraca	Mysida	Mysidae	*Caspiomysis knipowitschi*	-	EU233622	CRUST
Malacostraca	Euphausiacea	Euphausiidae	*Euphausia superba*	antartic krill	AMP19648.1	OP2/MWS
Malacostraca	Euphausiacea	Euphausiidae	*Euphausia superba*	antartic krill	AMP19649.1	OP3/MWS
Malacostraca	Euphausiacea	Euphausiidae	*Euphausia superba*	antartic krill	AMP19650.1	OP4/MWS
Malacostraca	Euphausiacea	Euphausiidae	*Euphausia superba*	antartic krill	AMP19651.1	OP5/MWS
Malacostraca	Euphausiacea	Euphausiidae	*Euphausia superba*	antartic krill	AMP19652.1	OP6/LWS
Malacostraca	Euphausiacea	Euphausiidae	*Euphausia superba*	antartic krill	DQ852580	CRUST
Malacostraca	Decapoda	Varunidae	*Hemigrapsus sanguineus*	Asian shore crab	BAA09132	Rh1/BCRa
Malacostraca	Decapoda	Varunidae	*Hemigrapsus sanguineus*	Asian shore crab	BAA09133	Rh2/BCRb
Malacostraca	Mysida	Mysidae	*Hemimysis anomala*	opossum shrimp	EU233620	CRUST
Malacostraca	Mysida	Mysidae	*Holmesimysis costata*	-	ABI48880.1	CRUST
Malacostraca	Decapoda	Nephropidae	*Homarus gammarus*	European lobster	DQ852590	CRUST
Malacostraca	Mysida	Mysidae	*Katamysisi warpachowsky*	opossum shrimp	EU233617	CRUST
Malacostraca	Decapoda	Ocypodidae	*Leptuca pugilator*	Atlantic sand fiddler crab	ADQ01809	Rh1/MW1
Malacostraca	Decapoda	Ocypodidae	*Leptuca pugilator*	Atlantic sand fiddler crab	ADQ01810	Rh2/MW1
Malacostraca	Decapoda	Ocypodidae	*Leptuca pugilator*	Atlantic sand fiddler crab	ADQ01811	Rh3/SW
Malacostraca	Mysida	Mysidae	*Mysis diluviana*	opossum shrimp	DQ852591	CRUST
Malacostraca	Mysida	Mysidae	*Mysis segerstralei*	opossum shrimp	EU233619	CRUST
Malacostraca	Stomatopoda	Gonodactylidae	*Neogonodactylus oerstedii*	rock mantis shrimp	ABG37008	Rh2/CRUST
Malacostraca	Stomatopoda	Gonodactylidae	*Neogonodactylus oerstedii*	rock mantis shrimp	ABG37009	Rh3/CRUST
Malacostraca	Stomatopoda	Gonodactylidae	*Neogonodactylus oerstedii*	rock mantis shrimp	ABG37007.1	Rh1/LMS
Malacostraca	Stomatopoda	Gonodactylidae	*Neogonodactylus oerstedii*	rock mantis shrimp	AIF73507.1	UV1
Malacostraca	Stomatopoda	Gonodactylidae	*Neogonodactylus oerstedii*	rock mantis shrimp	AIF73508.1	UV2
Malacostraca	Stomatopoda	Gonodactylidae	*Neogonodactylus oerstedii*	rock mantis shrimp	AIF73509.1	UV3
Malacostraca	Stomatopoda	Gonodactylidae	*Neogonodactylus oerstedii*	rock mantis shrimp	AUC64077.1	LWS1
Malacostraca	Stomatopoda	Gonodactylidae	*Neogonodactylus oerstedii*	rock mantis shrimp	AUC64078.1	MWS
Malacostraca	Stomatopoda	Gonodactylidae	*Neogonodactylus oerstedii*	rock mantis shrimp	AUC64079.1	LWS2

Malacostraca	Stomatopoda	Gonodactylidae	*Neogonodactylus oerstedii*	rock mantis shrimp	AUC64080.1	LWS3
Malacostraca	Stomatopoda	Gonodactylidae	*Neogonodactylus oerstedii*	rock mantis shrimp	ACU00223	rOp4 /LW2
Malacostraca	Stomatopoda	Gonodactylidae	*Neogonodactylus oerstedii*	rock mantis shrimp	ACU00224	rOp5 /LW2
Malacostraca	Stomatopoda	Gonodactylidae	*Neogonodactylus oerstedii*	rock mantis shrimp	ACU00222	rOp6 /LW2
Malacostraca	Stomatopoda	Gonodactylidae	*Neogonodactylus oerstedii*	rock mantis shrimp	ACU00210	rOp7 /LW2
Malacostraca	Stomatopoda	Gonodactylidae	*Neogonodactylus oerstedii*	rock mantis shrimp	ACU00211	rOp8 /LW2
Malacostraca	Stomatopoda	Gonodactylidae	*Neogonodactylus oerstedii*	rock mantis shrimp	ACU00218	rOp9 /LW2
Malacostraca	Stomatopoda	Gonodactylidae	*Neogonodactylus oerstedii*	rock mantis shrimp	ACU00217	rOp10 /LW2
Malacostraca	Stomatopoda	Gonodactylidae	*Neogonodactylus oerstedii*	rock mantis shrimp	ACU00221	rOp11 /LW2
Malacostraca	Stomatopoda	Gonodactylidae	*Neogonodactylus oerstedii*	rock mantis shrimp	ACU00216	rOp12 /LW2
Malacostraca	Stomatopoda	Gonodactylidae	*Neogonodactylus oerstedii*	rock mantis shrimp	ACU00215	rOp13 /LW2
Malacostraca	Stomatopoda	Gonodactylidae	*Neogonodactylus oerstedii*	rock mantis shrimp	ACU00212	rOp14 /LW2
Malacostraca	Stomatopoda	Gonodactylidae	*Neogonodactylus oerstedii*	rock mantis shrimp	ACU00220	rOp15 /LW2
Malacostraca	Stomatopoda	Gonodactylidae	*Neogonodactylus oerstedii*	rock mantis shrimp	ACU00219	rOp16 /LW2
Malacostraca	Stomatopoda	Gonodactylidae	*Neogonodactylus oerstedii*	rock mantis shrimp	ACU00214	rOp17 /LW2
Malacostraca	Stomatopoda	Gonodactylidae	*Neogonodactylus oerstedii*	rock mantis shrimp	ACU00213	rOp18 /LW2
Malacostraca	Mysida	Mysidae	*Neomysis americana*	opossum shrimp	DQ852598.1	CRUST
Malacostraca	Mysida	Mysidae	*Paramysis intermedia*	opossum shrimp	EU233609	CRUST
Malacostraca	Decapoda	Cambaridae	*Procambarus clarkii*	Louisiana crawfish	ALJ26467.1	LWS
Malacostraca	Decapoda	Cambaridae	*Procambarus clarkii*	Louisiana crawfish	ALJ26468.1	SWS
Malacostraca	Decapoda	Cambaridae	*Procambarus clarkii*	Louisiana crawfish	S53494	LWS
Malacostraca	Decapoda	Oplophoridae	*Janicella spinicauda*	Bioluminescent shrimp	MN939402 (Jspi_E_DN16415_c0_g1_i1)	SWS2
Malacostraca	Decapoda	Oplophoridae	*Janicella spinicauda*	Bioluminescent shrimp	MN939400 (Jspi_E_DN7379_c0_g1_i1)	MWS1 (eye)
Malacostraca	Decapoda	Oplophoridae	*Janicella spinicauda*	Bioluminescent shrimp	MN939404 (Jspi_P_DN45546_c0_g1_i1)	MWS1 (photophore)
Malacostraca	Decapoda	Oplophoridae	*Janicella spinicauda*	Bioluminescent shrimp	MN939401 (Jspi_E_DN4693_c0_g1_i1)	MWS2 (eye)
Malacostraca	Decapoda	Oplophoridae	*Janicella spinicauda*	Bioluminescent shrimp	MN939405 (Jspi_P_DN31556_c0_g1_i1)	MWS2 (photophore)
Malacostraca	Decapoda	Oplophoridae	*Janicella spinicauda*	Bioluminescent shrimp	MN939403 (Jspi_P_DN14462_c0_g1_i1)	LWS2 (photophore)

Malacostraca	Decapoda	Oplophoridae	*Janicella spinicauda*	Bioluminescent shrimp	MN939399 (Jspi_E_DN11104_c0_g1_i1)	LWS2a
Malacostraca	Decapoda	Oplophoridae	*Janicella spinicauda*	Bioluminescent shrimp	MN939398 (Jspi_E_DN7709_c0_g1_i1_ext)	LWS2b
Malacostraca	Mysida	Mysidae	*Schistomysis assimilis*	-	EU233621	CRUST
Merostomata	Xiphosurida	Limulidae	*Limulus polyphemus*	Atlantic horseshoe crab	ACO05013	Op5/BCR
Merostomata	Xiphosurida	Limulidae	*Limulus polyphemus*	Atlantic horseshoe crab	AAA02498	Ops1/LMS
Merostomata	Xiphosurida	Limulidae	*Limulus polyphemus*	Atlantic horseshoe crab	AAA02499	Ops2/LMS2
Merostomata	Xiphosurida	Limulidae	*Limulus polyphemus*	Atlantic horseshoe crab	AAO59500	Ops3/LMS3
Merostomata	Xiphosurida	Limulidae	*Limulus polyphemus*	Atlantic horseshoe crab	P35360	Ops4/LMS4
Merostomata	Xiphosurida	Limulidae	*Limulus polyphemus*	Atlantic horseshoe crab	AIT75830	Ops6/LW1
Merostomata	Xiphosurida	Limulidae	*Limulus polyphemus*	Atlantic horseshoe crab	AIT75831	Ops7/LW1
Merostomata	Xiphosurida	Limulidae	*Limulus polyphemus*	Atlantic horseshoe crab	AIT75832	Ops8/LW2
Merostomata	Xiphosurida	Limulidae	*Limulus polyphemus*	Atlantic horseshoe crab	AEL29244	UV
Ostracoda	Myodocopida	Cypridinidae	*Skogsbergia lerneri*	-	AAL37539	SlCE10#/MW2
Ostracoda	Myodocopida	Cypridinidae	*Skogsbergia lerneri*	-	AAL37521	SlME27#/MW2
Ostracoda	Myodocopida	Cypridinidae	*Vargula hilgendorfii*	sea firefly	AAL37507	VhCE7#/MW2
Ostracoda	Myodocopida	Cypridinidae	*Vargula hilgendorfii*	sea firefly	AAL37513	VhME16#/MW2
Ostracoda	Myodocopida	Cypridinidae	*Vargula tsujii*	California sea firefly	ADZ45236	Vtops /MW2
Other (Annelida)	Rhynchobdellida	Glossiphoniidae	*Helobdella robusta*	leech	Genome	MEL1
Other (Annelida)	Rhynchobdellida	Glossiphoniidae	*Helobdella robusta*	leech	Genome, scaffold_391	MEL2
Other (Cephalopoda)	n/a	Ommastrephidae	*Todarodes pacificus*	Japanese flying squid	X70498	LWS
Other (Echinoidea)	Echinoida	Strongylocentrotidae	*Strongylocentrotus purpuratus*	purple sea urchin	XR_026330	MEL1
Other (Echinoidea)	Echinoida	Strongylocentrotidae	*Strongylocentrotus purpuratus*	purple sea urchin	DQ285097	MEL2
Other (Gastropoda)	n/a	Aplysiidae	*Aplysia californica*	California seahare	AASC01108363	MEL1
Other (Gastropoda)	n/a	Aplysiidae	*Aplysia californica*	California seahare	AASC02005512	MEL2
Other (Gastropoda)	n/a	Lottiidae	*Lottia gigantea*	owl limpet	FC774055	MEL1
Other (Gastropoda)	n/a	Lottiidae	*Lottia gigantea*	owl limpet	Genome	MEL2
Other (Platyhelminthes)	Tricladida	Dugesiidae	*Dugesia japonica*	flatworm	CAD13146	MELL
Other (Platyhelminthes)	Tricladida	Dugesiidae	*Girardia tigrina*	flatwom	CAB89516	MELL
Other (Platyhelminthes)	Diplostomida	Schistosomatidae	*Schistosoma mansoni*	blood fluke	AF155134	MEL1
Other (Platyhelminthes)	Diplostomida	Schistosomatidae	*Schistosoma mansoni*	blood fluke	CD096414	MEL2
Other (Platyhelminthes)	Diplostomida	Schistosomatidae	*Schistosoma mansoni*	blood fluke	Smp_180030	MEL3

Other (Platyhelminthes)	Tricladida	Dugesiidae	*Schmidtea mediterranea*	flatworm	AF112361	MEL1
Other (Platyhelminthes)	Tricladida	Dugesiidae	*Schmidtea mediterranea*	flatworm	AAD28720.1	MELL
Other (Polychaeta)	n/a	Capitellidae	*Capitella capitata*	polychaete	Genome	MEL1
Other (Polychaeta)	Phyllodocida	Nereididae	*Platynereis dumerilii*	marine annelid	AJ316544	MEL1
